# Regulatory Standards and Guidance for the Use of Health Apps for Self-Management in Sub-Saharan Africa: Scoping Review

**DOI:** 10.2196/49163

**Published:** 2024-04-11

**Authors:** Benard Ayaka Bene, Sunny Ibeneme, Kayode Philip Fadahunsi, Bala Isa Harri, Nkiruka Ukor, Nikolaos Mastellos, Azeem Majeed, Josip Car

**Affiliations:** 1 Department of Primary Care and Public Health School of Public Health Imperial College London London United Kingdom; 2 Department of Public Health Federal Ministry of Health Abuja Nigeria; 3 Digital Health Specialist UNICEF East Asia Pacific Regional Office Bangkok Thailand; 4 Department of Health Planning, Research and Statistics Federal Ministry of Health Abuja Nigeria; 5 Strategic Health Information Cluster World Health Organization Abuja Nigeria; 6 School of Life Course & Population Sciences King’s College London London United Kingdom

**Keywords:** regulation, standard, framework, guidance, guideline, health app, self-management, World Health Organization, WHO African Region, sub-Saharan Africa

## Abstract

**Background:**

Health apps are increasingly recognized as crucial tools for enhancing health care delivery. Many countries, particularly those in sub-Saharan Africa, can substantially benefit from using health apps to support self-management and thus help to achieve universal health coverage and the third sustainable development goal. However, most health apps published in app stores are of unknown or poor quality, which poses a risk to patient safety. Regulatory standards and guidance can help address this risk and promote patient safety.

**Objective:**

This review aims to assess the regulatory standards and guidance for health apps supporting evidence-based best practices in sub-Saharan Africa with a focus on self-management.

**Methods:**

A methodological framework for scoping reviews was applied. A search strategy was built and applied across the following databases, gray literature sources, and institutional websites: PubMed, Scopus, World Health Organization (WHO) African Index Medicus, OpenGrey, WHO Regional Office for Africa Library, ICTworks, WHO Directory of eHealth policies, HIS Strengthening Resource Center, International Telecommunication Union, Ministry of Health websites, and Google. The search covered the period between January 2005 and January 2024. The findings were analyzed using a deductive descriptive content analysis. The policy analysis framework was adapted and used to organize the findings. The Reporting Items for Stakeholder Analysis tool guided the identification and mapping of key stakeholders based on their roles in regulating health apps for self-management.

**Results:**

The study included 49 documents from 31 sub-Saharan African countries. While all the documents were relevant for stakeholder identification and mapping, only 3 regulatory standards and guidance contained relevant information on regulation of health apps. These standards and guidance primarily aimed to build mutual trust; promote integration, inclusion, and equitable access to services; and address implementation issues and poor coordination. They provided guidance on systems quality, software acquisition and maintenance, security measures, data exchange, interoperability and integration, involvement of relevant stakeholders, and equitable access to services. To enhance implementation, the standards highlight that legal authority, coordination of activities, building capacity, and monitoring and evaluation are required. A number of stakeholders, including governments, regulatory bodies, funders, intergovernmental and nongovernmental organizations, academia, and the health care community, were identified to play key roles in regulating health apps.

**Conclusions:**

Health apps have huge potential to support self-management in sub-Saharan Africa, but the lack of regulatory standards and guidance constitutes a major barrier. Hence, for these apps to be safely and effectively integrated into health care, more attention should be given to regulation. Learning from countries with effective regulations can help sub-Saharan Africa build a more robust and responsive regulatory system, ensuring the safe and beneficial use of health apps across the region.

**International Registered Report Identifier (IRRID):**

RR2-10.1136/bmjopen-2018-025714

## Introduction

### Background

Health apps are the most widely used digital health products globally [1,2]. Harnessing the potential of health apps creates a huge opportunity in providing support for health care delivery, including patient communication, patient education, and decision support for self-management [3-8]. Health apps can be an effective tool to strengthen health systems worldwide, especially in low- and middle-income countries including those in sub-Saharan Africa [4,5,9]. As a result, the attainment of universal health coverage (UHC) and sustainable development goal (SDG) 3, good health and well-being, can be accelerated [8,10].

Many health apps fall below the expected quality threshold [11]. Several studies have found that widely used health apps are often technically unreliable and clinically unsafe [12-14] and do not comply with ethical standards and the principles of confidentiality of information and data privacy [15,16]. In addition, many commercially available health apps were not developed using interoperability standards that are widely accepted in sub-Saharan Africa (eg, Fast Healthcare Interoperability Resources [FHIR]) [17-20]. Consequently, it becomes difficult to integrate these apps into a clinical workflow.

Hence, regulation through robust mechanisms is crucial to enhance the development, implementation, and adoption of health apps. Regulatory standards and guidance are essential for the safety of patients as they ensure quality assurance of any new technology in health care and contribute to building mutual trust while promoting the optimal use of the technology [21-23]. Therefore, to ensure that health apps that are used to support the self-management of patients are technically reliable and clinically safe, interoperable across systems, and compliant with the principles of confidentiality of information and data privacy, there is a need for effective regulatory standards. Furthermore, effective regulation can help ensure that health apps for self-management are culturally functional and competent and are accessible to those who need them regardless of gender, ethnicity, geographical location, or financial status [24-31].

Since 2005, there have been ongoing efforts to strengthen digital health governance at both the national and international levels [32,33]. In 2018, the World Health Organization (WHO) member states renewed their commitment to using digital health technologies (DHTs) to advance UHC and SDG 3 [33]. However, to date, the extent to which the use of health apps for self-management is regulated across countries within the WHO African Region (also known as sub-Saharan Africa) remains unclear. Therefore, this review was conducted to identify available regulatory standards and guidance and assess the extent to which they regulate health apps for self-management in sub-Saharan Africa. The review also mapped out the key stakeholders and their roles in regulating health apps for self-management across sub-Saharan Africa.

### Review Questions

The review attempted to answer the following questions: (1) What regulatory standards and guidance are available for regulating health apps for self-management across sub-Saharan Africa? (2) To what extent do regulatory standards and guidance regulate health apps for self-management in terms of what aspects are regulated; why, how, and for whom; and what aspects are not regulated? (3) Who are the key stakeholders and what are their roles in regulating health apps for self-management?

## Methods

### Study Design

The process of this scoping review followed the methodological framework for conducting a scoping study originally described by Arksey and O’Malley [34] and the updated methodological guidance for conducting a Joanna Briggs Institute scoping review [34-37]. The reporting of the review was guided by the PRISMA-ScR (Preferred Reporting Items for Systematic Reviews and Meta-Analyses extension for Scoping Reviews) checklist [38]. A completed PRISMA-ScR checklist is provided in Multimedia Appendix 1. The protocol of this scoping review was published in *BMJ Open* [30].

### Identifying Relevant Documents

Two reviewers (BAB and SI) developed the search strategy with the assistance of a librarian and in consultation with other research team members (KPF, BIH, NU, NM, AM, and JC). The following key terms were included: policy, legislation, strategy, regulation, standard, criterion, framework, guidance, guideline, digital health, eHealth, app, WHO African Region, and sub-Saharan Africa, and the names of all sub-Saharan African countries.

Owing to the absence of regulatory standards and guidance in scientific databases, the search focus was narrowed down to gray literature sources and institutional websites, including OpenGrey, WHO Regional Office for Africa (AFRO) Library, repositories for digital health policies (ICTworks, WHO’s Directory of eHealth Policies, and Health Information System Strengthening Resource Center), as well as the websites of WHO, International Telecommunication Union (ITU), and Ministries of Health (MOHs). The only scientific databases searched were PubMed, Scopus, and WHO AIM. PubMed was not included in the protocol. We also conducted a systematic search on Google. We used truncation to increase the yield of the results. The search strategy was then applied across PubMed, Scopus, and WHO AIM databases using Boolean terms (mainly *OR* and *AND*) to combine search results. Gray literature sources and institutional websites were searched using phrases containing ≥2 keywords such as “eHealth regulation,” “digital health regulatory standard,” “eHealth regulatory standard,” “digital health regulation,” “digital health policy,” “eHealth policy,” “digital health strategy,” and “eHealth strategy.” For Google search, we added the names of the country to the phrases (eg, “digital health regulation Nigeria”). The reference lists of the included documents were also searched, and key individuals at the MOHs, WHO Country Offices, and the WHO AFRO were contacted for related documents. When our search was conducted, the WHO Directory of eHealth policies website was unavailable, and the WHO AFRO Library was undergoing reconstruction. The search strategies for PubMed, Scopus, and WHO AIM are provided in Multimedia Appendix 2. The search was conducted between 2005 and January 2024.

### Study Selection

The search results obtained from PubMed, Scopus, and WHO AIM were imported into Mendeley (Elsevier) [39] to remove duplicates. The search conducted on OpenGrey did not yield any results, whereas relevant records obtained from institutional websites, repositories, and Google were downloaded as PDF copies and uploaded to Mendeley. After removing duplicates, the remaining results were imported into Covidence (Veritas Health Innovation) [40] for screening. Two reviewers (BAB and SI) applied the predefined eligibility criteria (Textbox 1) to screen the documents in 2 stages (title and abstract or executive summary). All discrepancies were discussed until the reviewers reached agreement.

**Inclusion and exclusion criteria.**

**Inclusion criteria**

- Type of document: Regulatory standards, guidance, policies, strategies, and committee or government reports that address regulatory issues related to the use of health apps for self-management
- Location: Documents developed and implemented in countries within sub-Saharan Africa
- Date of publication: Documents developed since 2005; the global efforts toward promoting standards to minimize variability and potential harms that could arise from poorly regulated use of digital health began in 2005 [33]
- Language: Documents written in English language and other official languages of sub-Saharan African countries (Portuguese and French)

**Exclusion criteria**

- Type of document: Standards, guidance, policies, strategies, and reports not related to regulation of health apps
- Location: Documents from countries outside sub-Saharan Africa
- Date of publication: Documents developed before 2005
- Language: None

### Data Charting (Extraction)

Two reviewers (BAB and SI), in consultation with the other members of the research team, developed the data extraction forms using an iterative process that included piloting data extraction and refinement until a consensus was reached.

We proposed in the study protocol [30] that data extraction would be conducted by the 2 reviewers independently. However, owing to the approach adopted for data extraction (deductive qualitative content analysis), 1 reviewer, rather than 2, initially extracted data from the included documents, and any concerns were discussed with a second reviewer [41]. Unresolved issues were then discussed and resolved with a third reviewer in a steering group meeting.

### Collating, Summarizing, and Reporting Results

To address the research questions (particularly question 2), we adopted a deductive descriptive qualitative content analysis method to analyze and report the key findings. The policy analysis framework by Walt and Gilson [42] was adapted and applied to ensure that there was a consistent way of organizing the key findings: (1) Content (which aspects are regulated and which aspects are not?)—these are the components that directly or indirectly address regulatory issues related to the use of health apps for self-management, including areas that have not been addressed. (2) Context (why are those aspects regulated?)—this characterizes the rationale indicated for addressing regulatory issues related to the use of health apps for self-management. (3) Process (how are the regulatory standards developed and implemented?)—this describes the methods or approaches used to develop and implement regulatory standards. (4) Actors (who are the regulatory standards targeted toward?)—these are the key actors targeted by the standards.

Using a deductive descriptive qualitative content analysis approach, we examined each included document to systematically identify texts for concepts, patterns, and other relevant information. We then categorized them under *content, context, process,* or *actors* in relation to regulating health apps for self-management. The findings under content and context were further organized based on 4 predefined regulatory categories or themes as documented in the literature, namely (1) *technical and clinical safety* [12-14], (2) *data protection and security* [15,16], (3) *standards and interoperability* [28,31], and (4) *inclusion and equitable access* [24-29].

To address the third research question, the Reporting Items for Stakeholder Analysis (RISA) tool [41] was used as a guide to group key stakeholders based on role categorization as recognized globally by the WHO, the ITU, and UNESCO [32,33,43].

### Ethical Considerations

Primary data were not collected in this study. Therefore, no ethics approval was required.

## Results

### Search Results

A total of 2900 records were obtained after removing duplicates. Although the literature search was conducted in English, the search also yielded documents written in French and Portuguese from the ICTworks repository [44]. Following the initial screening of the title and abstract (or executive summaries), 73 documents were retrieved for full-text assessment. After applying the inclusion criteria for the full-text assessment, 49 documents were found eligible for inclusion in the review.

**Figure 1:**
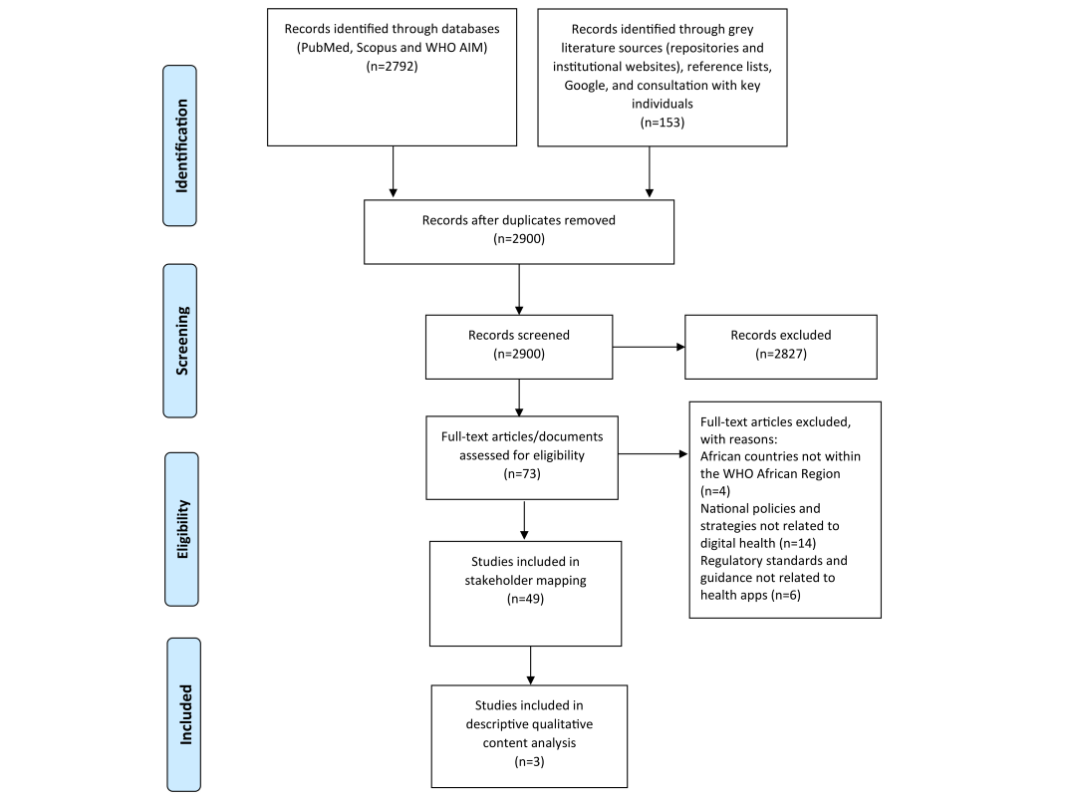
PRISMA (Preferred Reporting Items for Systematic Reviews and Meta-Analyses) flow diagram showing the study selection process. The descriptive qualitative content analysis included only 3 of the 49 (6%) documents used for stakeholder mapping. WHO: World Health Organization.

The PRISMA (Preferred Reporting Items for Systematic Reviews and Meta-Analyses) flow diagram [45] showing the study selection process is presented in Figure 1.

### Types of Documents

On the basis of the inclusion criteria, 3 categories of documents were considered for this review, namely “stand-alone regulatory standards and guidance that potentially regulate health apps for self-management,” “national policies and strategies on digital health,” and “other national documents that relate to the regulation of health apps for self-management.” Table 1 presents the types of documents obtained for each country within sub-Saharan Africa.

**Table 1:** Types of documents obtained for each sub-Saharan African country.

Country	Type of document		
	Stand-alone regulatory standards and guidance	National policies and strategies on digital health	Other related national documents
Algeria			
Angola			
Benin		✓	
Botswana		✓	
Burkina Faso		✓	
Burundi		✓	
Cameroon		✓	
Cape Verde			
Central African Republic			
Chad			
Comoros		✓	
Côte d’Ivoire (Ivory Coast)		✓	
Democratic Republic of the Congo		✓	
Equatorial Guinea			
Eritrea			
Eswatini		✓	
Ethiopia	✓	✓	✓
Gabon		✓	
Gambia		✓	
Ghana		✓	
Guinea			
Guinea-Bissau			
Kenya	✓	✓	✓
Lesotho		✓	
Liberia		✓	✓
Madagascar		✓	
Malawi		✓	
Mali		✓	
Mauritania			
Mauritius		✓	
Mozambique		✓	
Namibia			
Niger		✓	
Nigeria	✓	✓	✓
Republic of the Congo (Congo Brazzaville)			
Rwanda		✓	
São Tomé and Príncipe			
Senegal		✓	
Seychelles			
Sierra Leone			
South Africa		✓	✓
South Sudan			
Tanzania		✓	✓
Togo		✓	
Uganda		✓	
Zambia		✓	
Zimbabwe		✓	

### Characteristics of the Included Documents

#### Stand-Alone Regulatory Standards and Guidance

We identified and included 6 stand-alone regulatory standards [18,19,46-49] from 3 countries (Ethiopia, Kenya, and Nigeria). All 6 documents were written in English. The years of development ranged between 2013 and 2021, as indicated in Multimedia Appendix 3. The years of implementation were not specifically stated.

Although none of the included regulatory standards were exclusively developed to regulate health apps for self-management, 3 of them (Kenya Standards and Guidelines for mHealth Systems [18], Kenya Standards and Guidelines for E-Health Systems Interoperability [47], and Health Sector Information and Communications Technology Standards and Guidelines [48]) provided concept and information relevant to the regulation of health apps and were included in the qualitative content analysis. The Kenya Standards and Guidelines for mHealth Systems [18] provides standards and guidelines on the design, development, and implementation of mobile health (mHealth) solutions to ensure they are interoperable, scalable, and sustainable. The Kenya Standards and Guidelines for E-Health Systems Interoperability [47] outlines the principles, requirements, and standards for eHealth systems interoperability in Kenya. The Health Sector Information and Communications Technology Standards and Guidelines [48] provide guidance and a consistent approach across the health sector in Kenya for establishing, acquiring, and maintaining current and future information systems and information and communications technology (ICT) infrastructure that foster interoperability across systems. These 3 documents are a good combination of regulatory standards and guidance that provide content and context relevant to the regulation of health apps in sub-Saharan Africa.

The remaining 3 standards (standard for electronic health record [EHR] system in Ethiopia [19], standards and guidelines for electronic medical record systems in Kenya [46], and the health information exchange standard operating procedure and guideline [49]) were exclusively developed for EHRs or electronic medical records. However, they contain information relevant for mapping stakeholders with potential roles in regulating health apps for supporting self-management.

#### National Policies and Strategies on Digital Health

This review includes 35 national policies and strategies that are related to digital health (potentially covering health apps) [50-84] from 31 countries written in English, French, and Portuguese (Benin, Botswana, Burkina Faso, Burundi, Cameroon, Comoros, Côte d’Ivoire [Ivory Coast], Democratic Republic of the Congo, Eswatini, Ethiopia, Gabon, Ghana, Kenya, Liberia, Madagascar, Malawi, Mali, Mauritius, Mozambique, Namibia, Niger, Nigeria, Rwanda, Senegal, Sierra Leone, South Africa, Tanzania, Togo, Uganda, Zambia, and Zimbabwe). Although the literature search was conducted in English, it also yielded documents written in French and Portuguese from the ICTworks repository. The years of development and implementation range between 2005 and 2030. Policies and strategies written in French and Portuguese were translated into English using Google Translate. Documents labeled as national development plans, strategic plans, and strategic development plans were considered as national strategies.

National policies and strategies do not offer specific standards or guidance, but rather outline the country’s vision, policy directions, and strategies for using digital technologies in health care. They provide useful information for identifying digital health stakeholders who can play a role in regulating health apps for self-management. For example, Nigeria has a separate National Digital Health Policy [72] and a National Digital Health Strategy [71]. Both documents were developed by building on the lessons learned from the end-term evaluation of the previous National Health ICT Strategic Framework [85]. They describe Nigeria’s renewed vision, mission, goals, objectives, and strategies for the development and implementation of digital health with the aim to improve the quality, efficiency, and effectiveness of health service delivery and health outcomes.

It is worth noting that for countries with >1 policy or strategy, we included only the most recent versions. For instance, as mentioned earlier, Nigeria now has both a national digital health policy and a national digital health strategy. These 2 documents supersede and thus replace the old National Health ICT Strategic Framework [86]. Details of included documents are presented in Multimedia Appendix 3.

#### Other Related National Documents

We included 8 other documents [20,85,87-92] from 6 countries (Ethiopia, Kenya, Liberia, Nigeria, South Africa, and Tanzania) that did not fall under either stand-alone regulatory standards and guidance or national policies and strategies. These were mostly frameworks, road maps, and reports that potentially provide information relevant to the use of health apps. The years of development and implementation range from 2016 to 2025. These documents do not provide standards or guidance, but they contain information that can help map the digital health stakeholders that potentially play a role in regulating health apps for self-management. When multiple versions of a document exist, only the latest version was taken into consideration. Multimedia Appendix 3 provides details of the included documents.

#### Content: Aspects That Are Regulated and Aspects That Are Not

##### Technical and Clinical Safety

**Figure 2:**
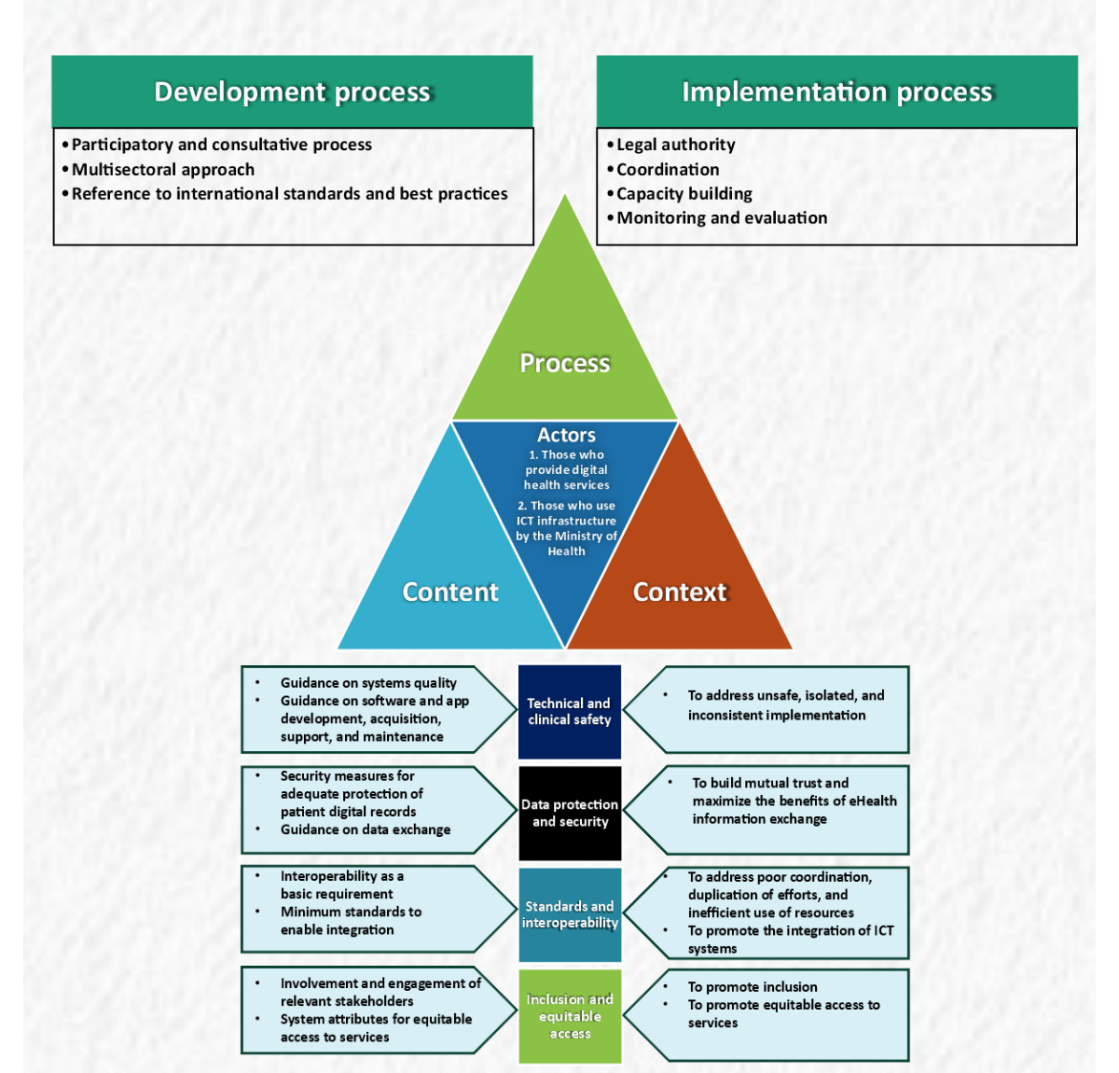
Summary of themes and subthemes covering content, context, process, and actors of the regulatory standards. The process is divided into development and implementation process. The subthemes relating to content and context are further categorized under technical and clinical safety, data protection and security, standards and interoperability, and inclusion and equitable access to services. ICT: information and communications technology.

Technical and clinical safety standards are required to prevent or minimize the harm that may arise from the use of the health ICT systems (including mHealth systems) as well as to improve the health outcomes and user satisfaction. As shown in Figure 2, two subthemes were generated from included standards [18,47,48] as content under technical and clinical safety: v(1) guidance on system quality and (2) guidance on software or app development, acquisition, support, and maintenance.

Notably, 2 of the included standards [18,47] provide guidance on system quality to ensure the quality, security, reliability, performance, and maintenance of eHealth and mHealth systems. The Kenya Standards and Guidelines for E-Health Systems Interoperability [47] recommend the implementation of a data quality protocol to ensure that the data collection, collation, analysis, interpretation, dissemination, and use are managed in accordance with the quality standards. Similarly, the Kenya Standards and Guidelines for mHealth Systems [18] recommends the inclusion of the following requirements in the technical manual: (1) minimum hardware requirements that should incorporate the preferred hardware architecture, (2) minimum software requirements that should include the minimum version of the underlying operating system as well as acceptable versions of related software, and (3) a detailed list of software dependencies (external libraries) necessary for the system to function properly.

The included standards [18,48] cover guidance on software or app development, acquisition, support, and maintenance, which aim to ensure the efficiency and effectiveness of eHealth and mHealth systems. The Kenya Standards and Guidelines for mHealth Systems [18] recommends a technical manual to provide a detailed description of the system’s installation and maintenance processes for system administrators and implementers; a developer’s guide for software developers and programmers to provide them with an overview of the system, description of the software design methodologies, description of the system architecture, and technical design diagrams; and a user manual to aid users in understanding how the system works and how each feature operates; in addition, the technical manual contains instructions for operating the software; entering and updating data; and generating, saving, and printing reports.

Although the contents generated here provide guidance that is relevant to health apps, they are not specific to health apps. Moreover, there are no clear measures to enable individuals or organizations that use health apps to manage clinical risk appropriately.

##### Data Protection and Security

Data protection and security are crucial aspects of managing patient information, thus ensuring the confidentiality, integrity, and availability of data as well as the rights and interests of the patient. Two subthemes related to data protection and security are (1) security measures for adequate protection of patients’ digital records and (2) guidance on data exchange.

The included standards [18,48] provide security measures for eHealth or mHealth systems to ensure the adequate protection of digitally accessible patient records. These measures include authentication, accountability, identification, authorization, integrity, confidentiality, availability, security, administration, and audit. This will help to achieve confidentiality, integrity, availability, and nonrepudiation of patient data or health records. Additional levels of security such as data encryption are required when there is a need to store sensitive information on removable devices or media or outside the MOH premises.

The Kenya Standards and Guidelines for mHealth Systems [18] provide the following guidance on data exchange to ensure privacy: (1) anonymize client data as much as possible before they can be shared; (2) where possible, use pseudonyms for the client data before they can be shared; (3) aggregate client data before they can be shared to reduce possibilities of tracing the data back to the client; and (4) minimize data so that access is available only to the data set required for that particular use. With regard to privacy rules, the Kenya Standards and Guidelines for E-Health Systems Interoperability [47] propose that a notice of privacy practices should be given to patients describing how their information may be used or shared while also specifying their legal rights.

##### Standards and Interoperability

Standards and interoperability are essential concepts in the field of IT, especially for systems that need to communicate and exchange data, as seen in the use of health apps for self-management. Two subthemes related to standards and interoperability are (1) interoperability as a basic requirement and (2) minimum standards to enable integration.

All the regulatory standards [18,47,48] highlight the importance of having interoperability as a basic requirement when selecting software products or services for use within the health system. This facilitates interaction across systems. For instance, to facilitate seamless interaction between mHealth systems and primary information systems for data capture, reporting, and decision support in various domains of the health system, the Kenya Standards and Guidelines for mHealth Systems [18] recommends the incorporation of at least 3 types of interoperability, namely, technical interoperability, semantic interoperability, and process interoperability.

Furthermore, 2 regulatory standards [18,47] proposed minimum interoperability standards to enable the integration of services and data exchange between various systems in health care. For instance, the Kenya Standards and Guidelines for mHealth Systems [18] suggests standards (for interoperability) for mHealth systems that are consistent with the recommendations in internationally accepted standards. They include the following: (1) clinical messaging—ensuring mHealth systems conform to Health Level 7 (HL7) version 3 standards and corresponding implementation guideline; (2) clinical terminology—ensuring terminologies and classifications for clinical concepts (eg, International Classification of Diseases, tenth revision—for diseases; Systemized Nomenclature of Medicine—for clinical data coding; Logical Observation Identifiers Names and Codes—for laboratories; and RxNorm—for Pharmacies); (3) the mHealth system must use the latest versions of international standards, such as HL7 Clinical Document Architecture for electronic sharing of clinical documents; (4) concepts—mHealth systems will use the idea of “concepts” so that information can be transmitted between systems without losing meaning or context, and HL7 Reference Implementation Model or other appropriate standards are recommended for implementing concepts; (5) architecture—to develop mHealth systems, developers should define the system architecture that should include data elements and business logic. Furthermore, to define how mHealth systems interact with other systems, developers of mHealth solutions must provide application programming interfaces. FHIR is the preferred application programming interface interoperability standard.

##### Inclusion and Equitable Access

Inclusion and equitable access are essential principles to ensure that health apps are culturally appropriate and relevant and accessible to everyone, regardless of gender, ethnicity, location, or economic status.

All the included regulatory standards [18,47,48] indicate that they were developed based on a combination of participatory and consultative approaches involving multiple actors or stakeholders, thus promoting inclusion. However, there are no specific measures or guidance to ensure adequate engagement and representation of all the relevant stakeholders and to sustain that engagement.

The Kenya Standards and Guidelines for mHealth Systems [18] proposes the following systems attributes to ensure equitable access to mHealth services at all times and from anywhere: (1) allocation of adequate storage and bandwidth capacity; (2) fast response time; (3) fast recovery capabilities; (4) performance monitoring; (5) business continuity processes, for example, backups; and (6) redundant sites and links. Furthermore, the Kenya Standards and Guidelines for mHealth Systems [18] prescribes the following metrics for measuring system availability: (1) downtime per year, (2) mean time between failure, (3) mean time to repair, and (4) failure in time.

Although the abovementioned systems attributes and metrics for measuring system availability are important, the included standards do not offer any concrete guidance or model for achieving a sustainable funding mechanism for health apps to ensure that they are readily available and accessible to those who need them.

#### Context: Reasons Why Those Aspects Are Regulated

##### Technical and Clinical Safety

The 3 standards [18,47,48] were developed to address unsafe, isolated, and inconsistent implementation. The Health Sector ICT Standards and Guidelines [48] suggest that although there has been a lot of ICT investment in the health sector leading to improvement in service delivery and information exchange, there remains the challenge of inconsistency in ICT implementation and harmonization of the health sector system requirements. Hence, there is a need to adopt global best practices for software development, acquisition, support, and maintenance by the MOH. In addition, the Kenya Standards and Guidelines for mHealth Systems [18] indicates that standards and guidelines are necessary to ensure a consistent approach to the development of ICT systems. Similarly, the Kenya Standards and Guidelines for E-Health Systems Interoperability [47] recognize the need to ensure that the processes of collecting, collating, analyzing, interpreting, disseminating, and using data are consistent with data quality standards.

##### Data Protection and Security

To build mutual trust and maximize the benefits of eHealth information exchange, the Kenya Standards and Guidelines for E-Health Systems Interoperability [47] reiterate that as health data are constantly being exchanged across health information systems, robust security standards are required to maintain their integrity and confidentiality. This will build the trust of service users and consequently help to maximize the benefits of eHealth information exchange such as in self-management.

##### Standards and Interoperability

Two of the included regulatory standards [47,48] indicate that the context for standards and interoperability was (1) to address poor coordination, duplication of efforts, and inefficient use of resources and (2) to promote the integration of ICT systems.

The Kenya Standards and Guidelines for E-Health Systems Interoperability [47] acknowledge that the absence of interoperability standards over the years has led to the duplication of efforts and the inefficient use of ICT resources in health care. Now that ICT has become increasingly relevant in improving efficiency in health service delivery, the Kenya MOH recognizes the need to adopt a standardized approach, hence the development of interoperability standards for eHealth systems. In addition, the Health Sector ICT Standards and Guidelines [48] emphasize the relevance of interoperability as a requirement for addressing the inconsistency in implementing ICT in the health sector.

The Health Sector ICT Standards and Guidelines [48] consider “integration of ICT systems” as one of its key guiding principles, acknowledging the lack of information systems integration as a challenge experienced by ICT services across Kenya.

##### Inclusion and Equitable Access

The contexts for inclusion and equitable access as generated from included standards [18,47,48] were (1) to promote inclusion and (2) to promote equitable access to services.

To promote inclusion, the standards [18,47,48] highlight the importance of involving and engaging multiple actors and stakeholders during the development process. However, no emphasis was placed on the need to sustain stakeholder engagement during the implementation process.

Pertaining to equitable access, the Kenya Standards and Guidelines for mHealth Systems [18] acknowledges that the public health care system is largely unavailable to most of the population in many developing countries because of geographical location, resource constraints, inefficiencies, and lack of awareness. Hence, it recognizes the importance of ensuring that mHealth services are always accessible by users and from anywhere as well as the need to put in place mechanisms to make this happen.

#### Process: How the Regulations Are Developed and Implemented

Two themes were generated from the included standards: development and implementation processes [18,47,48].

##### Development Process

All the included standards [18,47,48] indicate that they were developed through a participatory process and in consultation with a range of subject experts and interest groups. In addition, the standards [18,47,48] adopted a multisectoral approach to engage health-related stakeholders from government ministries or agencies and development partners and a range of subject experts and interest groups. It has also been reported that these standards [18,47,48] were developed based on international best practices and with reference to international standards. However, there is no indication that a stakeholder engagement strategy was adopted to sustain the engagement of stakeholders through the entire development and implementation process.

##### Implementation Process

The 3 regulatory standards [18,47,48] identify the key requirements to ensure effective implementation of IT services in the health sector. These are (1) legal authority, (2) coordination, (3) building capacity, and (4) monitoring and evaluation.

The included standards [18,47,48] were established based on the legal provisions enshrined in the health and other related acts and laws of Kenya as well as the relevant policies and strategies. Hence, it is expected that their implementation will comply with and be backed by those legal provisions. For example, the Health Sector ICT Standards and Guidelines [48] indicate that its implementation will be supported by the authority from the Kenya Communications Act 2009, E-Government Strategy, and National ICT Policy. Similarly, the Kenya Standards and Guidelines for mHealth Systems [18] asserts that it will be implemented by complying with existing and relevant national policies, legal frameworks, strategies, and standards, including the Health Information Policy, ICT Standards, and System Interoperability Principles.

The included standards [18,47,48] report that the implementation of regulations will require robust coordination mechanisms. For instance, the Health Sector ICT Standards and Guidelines [48] indicate that, as the Ministry’s ICT resource manager, the principal secretary (also the head of ICT), in collaboration with the ICT Governance Committee, is responsible for coordinating the implementation of the standard. The ICT Governance Committee comprises representatives from the heads of departments and ICT development partners in the health sector. The committee’s responsibilities include overseeing, enforcing, and reviewing standards as well as initiating ICT projects.

The Health Sector ICT Standards and Guidelines [48] highlight the need for capacity building or training of the MOH staff and stakeholders who are the primary users of the Ministry’s ICT services. This will enhance their capacity to implement the guidelines provided in the document in line with the ministry’s human resource development policies, regulations, and rules. However, it is acknowledged that building capacity for health ICT is a challenge given that there is low adoption of ICT among health providers, and ICT is not routinely included in the course content of most training programs. The Kenya Standards and Guidelines for mHealth Systems [18] listed the “number of mHealth practitioners trained on the standards and guidelines” as one of the indicators for monitoring and evaluating mHealth interventions.

The Health Sector ICT Standards and Guidelines [48] assert that monitoring and evaluation is an essential role of the MOH to ensure efficiency, accountability, and transparency throughout the implementation period. It further stresses that all those who use the Ministry’s ICT services are required to adhere to the provisions in the standard as the MOH will carry out quarterly monitoring exercises on the use of the standard to ensure compliance based on clear indicators. Furthermore, the ICT Governance Committee will periodically review and amend the standard to keep it relevant and effective. Similarly, the Kenya Standards and Guidelines for mHealth Systems [18] establishes the following key indicators for effectively monitoring and evaluating the implementation of the standards and guidelines: (1) the number of counties in which the MOH has disseminated the standards and guidelines, (2) the number of counties successfully implementing the standards and guidelines, (3) the number of mHealth practitioners trained on the standards and guidelines, (4) the number of mHealth practitioners accessing the standards and guidelines, (5) the number of mHealth practitioners who correctly understand the standards and guidelines, (6) the number of stakeholders who adhere to the standards and guidelines, (7) the number of mHealth systems that follow the required development steps, and (8) the number of mHealth practitioners who have implemented their systems by using the standards and guidelines. In addition, the Kenya Standards and Guidelines for mHealth Systems [18] indicates that the outlined standards will be reviewed every 3 years to ensure they are up to date with new changes including the changes in policies and systems upgrades.

Although all the abovementioned indicators are relevant, the implementation process is not explicit on the approach for regulating health apps and ensuring compliance with regulatory standards and guidance.

#### Actors: Those the Regulations Are Targeted at

The included standards [18,47,48] identified 2 main groups of actors for whom the regulations and guidance were targeted. They included (1) those who provide digital health services and (2) those who use the ICT infrastructure of the MOH.

Two of the standards [47,48] indicated that the regulations should be implemented by all individuals and organizations that provide ICT-related health care services to the public. Similarly, the Health Sector ICT Standards and Guidelines [48] state that all those who access or use the MOH ICT infrastructure are expected to adhere to the guidelines outlined in the document.

### Mapping of Stakeholders

To address the third research question, we conducted a stakeholder mapping guided by the RISA tool [41].

A total of 11 categories of key stakeholders were identified from all 49 included documents (6 stand-alone regulatory standards and guidance, 35 national policies or strategies, and 8 other related documents). These categories are consistent with the digital health stakeholders recognized by the WHO, ITU, and UNESCO [32,33,43]. Table 2 presents the mapping of stakeholders according to their role categorization. A more detailed table with a potential role description with regard to regulating health apps for self-management is presented in Multimedia Appendix 4.

**Table 2:** Mapping of stakeholders according to their potential role with regard to regulating health apps for self-management.

Stakeholder category	List of stakeholders	Role categorization
A1: Government (health sector)	Ministry of Health Relevant departments and agencies, including the National Medicines Regulatory Authority	Coordination and provision of an enabling environment
A2: Government (non–health sector)	Ministry of Power or Energy Ministry of Information and Communications Technology or Telecommunication Ministry of Education Ministry of Science and Technology Ministry of Finance Ministry of Justice	Coordination and provision of an enabling environment
B: Regulatory bodies	Relevant health regulatory agencies Ministry of Justice Law enforcement agencies	Compliance
C1: Funding bodies	Donors and aid agencies Foundations and development banks The private sector Other health care funders	Funding and insurance
C2: Insurance	The insurance industry	Funding and insurance
D1: Intergovernmental, international, and continental organizations	African Union WHO^a^ or WHO Regional Office for Africa International Telecommunication Union World Bank United Nations Children’s Fund	Strategic support
D2: Nonstate actors	Nongovernmental organizations Civil society organizations Faith-based organizations	Strategic support
E1: Industries and businesses that influence the use of health apps	App developers Network or internet providers App evaluators	Resources and skills
E2: Academia and research bodies and institutions	Universities Teaching hospitals Research institutes	Resources and skills
E3: Professionals in research and practice or	Subject matter experts	Resources and skills
F1: The health care community (providers)	Health care providers (eg, hospitals, clinics, and primary health cares) Health care professionals	Service delivery and use
F2: The health care community (users)	Patients Caregivers Families Community groups	Service delivery and use

^a^WHO: World Health Organization.

## Discussion

### Overview

This paper presents the findings of a scoping review of regulatory standards and guidance for the use of health apps for self-management in sub-Saharan Africa. To the best of our knowledge, this is the first study that attempted to identify and assess the extent to which regulatory standards and guidance regulate and guide the use of health apps for self-management in sub-Saharan Africa as well as map out the key stakeholders and their potential roles.

Our findings reveal that only 1 country (Kenya) in sub-Saharan Africa currently has national regulatory standards that could potentially regulate the use of health apps for self-management. The included standards failed to adequately address adequate attention to inclusion and equitable access. This is concerning given the growing need to promote the adoption of culturally appropriate and relevant health apps and to ensure that they are available to those who need them regardless of gender, ethnicity, geographical location, or financial status [24-29]. Consequently, this review provides insights into the regulation of health apps for self-management in sub-Saharan Africa, which needs to be given more attention if the potential of these apps is to be harnessed in the region.

### Principal Findings

We identified 49 documents from 31 countries in sub-Saharan Africa. Although none of the included standards provided a specific set of regulations on health apps for self-management, we identified 3 standards [18,47,48] that provided relevant information regarding the regulation of health apps. The included national policies and strategies, in contrast, only outline the goals and commitments made by national governments to promote the adoption of digital technologies in the health sector and the plans and paths set forth to achieve these goals. However, the information they provided was relevant for identifying and mapping digital health stakeholders who potentially have vital roles in regulating the use of health apps for self-management.

The policy analysis framework (content, context, process, and actors) [42] was adapted and applied to organize the key findings. The content covered the following areas: guidance on systems quality; guidance on software and app development, acquisition, support, and maintenance; security measures for adequate protection of patients’ digital records; guidance on data exchange; interoperability as a basic requirement; minimum standards to enable integration; involvement and engagement of relevant stakeholders; and system attributes for equitable access to services. Meanwhile, the context was to address unsafe, isolated, and inconsistent implementation; to build mutual trust and maximize the benefits of eHealth information exchange; to address poor coordination, duplication of efforts, and inefficient use of resources; to promote the integration of ICT systems; and to promote inclusion and equitable access to services. The process involved the development process (which covers participatory and consultative processes and multisectoral approach, with reference to international standards and best practices) and the implementation process (which covers legal authority, coordination, capacity building, and monitoring and evaluation). The targeted actors were those who provided digital health services and those who used the ICT infrastructure of the MOH.

Furthermore, key stakeholders with potential roles in regulating health apps for self-management were identified. They include the government, regulatory bodies, funders, intergovernmental and nongovernmental organizations, academia, and the health care community.

### Implications of the Study Findings for Practice

#### Overview

Regulatory standards and guidance act as a bridge between technological innovation and its safe and effective use in health care. They ensure that while technology continues to advance, the safety and trust of patients are never compromised. Among the plethora of health apps on the market, the over-the-counter, nonregulated apps such as wellness and fitness apps are the most mainstream [93-95]. On the other side of the spectrum, there are regulated health apps that are classified under medical devices or software as medical device products [94,95]. Some of these are prescription-only apps, such as digital therapeutics (DTx) apps for managing substance dependence [95,96].

Although some high-income countries have made significant strides in ensuring the safety, effectiveness, and accessibility of health apps, the journey has indeed not been without challenges and hurdles. Sub-Saharan Africa, although dealing with its own unique set of challenges, has the opportunity to learn from the experiences of these high-income countries. This could potentially allow the region to bypass some of the hurdles encountered by high-income countries in their journeys.

#### Technical and Clinical Safety

Technical and clinical safety are essential requirements that health apps must meet before they can be considered for use for self-management to minimize the risk of harm to patients. It is well documented that health apps that function poorly pose a serious threat to the safety of patients. An example illustrating how health apps used for self-management can threaten patient safety is evident in a study [12]. This study [12] revealed that widely used health apps designed to calculate and estimate insulin doses could endanger patients by providing incorrect or inappropriate dose recommendations. Similarly, 2 successive studies that assessed the contents and tools of apps for asthma discovered that none of the apps in the first study offered comprehensive information or adequate tools for asthma self-management, whereas the follow-up study, which was conducted 2 years later, showed a 2-fold increase in the number of asthma apps, yet there was no improvement in the content and tools offered by the newer apps. In fact, many apps recommended self-management procedures that were not supported by evidence [13,14]. Accordingly, some health apps that support the self-management of long-term conditions do not adhere to evidence-based guidelines and are unresponsive to the evolving health needs of patients.

Although the context of included regulatory standards with regard to technical and clinical safety was to address unsafe, isolated, and inconsistent implementation, the guidance provided by these regulatory standards is not specific to health apps, and they do not provide appropriate guidance and standards for health organizations and other key stakeholders to establish a framework for managing the clinical risks associated with deploying and implementing self-management health apps. Considering the rapid advancements in digital health (including artificial intelligence [AI] or machine learning and big data), health apps will increasingly play a crucial role in supporting self-management through digitally enabled care pathways that will improve personalized care and health outcomes [97,98]. Therefore, it is imperative to ensure the technical reliability and clinical safety of health apps for self-management through robust regulatory standards and guidance. For instance, a guide on the criteria for health app assessment, developed by the UK government, includes technical stability and clinical safety as criteria for deciding whether health apps should be considered for use in the National Health Service (NHS) [99]. In addition, medical device apps are required to conform to the NHS clinical risk management standards as part of the clinical safety requirements [99,100]. In the event of any concerns regarding the safety of a medical device app, the Yellow Card reporting system can be used by a responsible clinical safety officer or any other individual to notify the Medicines and Healthcare products Regulatory Agency (MHRA) [101,102].

#### Data Protection and Security

To adequately manage patient information when health apps are used for self-management, data protection and security standards and guidance are required. They guarantee that data are kept and handled safely and responsibly within the provisions of the law and that patients’ rights and interests are respected.

There have been ongoing concerns about compliance with ethical standards, the principles of confidentiality of information, and data privacy. For example, an assessment of apps that had previously been endorsed by the former UK NHS Apps Library revealed substantial gaps in compliance with data protection principles regarding the collection, storage, and transmission of personal information. This has raised a fundamental concern about the credibility of developer disclosures and whether these disclosures can be trusted by certification programs [15]. A study assessed the privacy practices of the 36 most popular apps for depression and smoking cessation for Android and iOS in the United States and Australia [16]. The findings revealed that although only 69% (25/36) of the apps included a privacy policy, 92% (33/36) of the apps shared data with a third party, and only 92% (23/25 with privacy policy) of the apps disclosed sharing data with a third party in their policy. Although 81% (29/36) of the apps shared data with Google and Facebook for the purposes of advertising, marketing, or analytics, only 43% (12/28) of the apps that shared data with Google and 50% (6/12) of the apps that shared data with Facebook disclosed this in their policy [16].

In this regard, health app developers and providers in the United Kingdom are required to conduct a data protection risk assessment before they launch or update their apps to ensure compliance with the United Kingdom General Data Protection Regulation (GDPR) and other relevant regulations, including the Data Protection Act 2018 [103]. By conducting a data protection risk assessment, health app developers and providers can demonstrate that they are accountable; they respect the privacy and dignity of their users; and that they deliver safe, effective, and ethical solutions [104].

Health apps are expected to play an increasingly important role in supporting self-management. However, this ambition can only be achieved if citizens trust that these apps are collecting and analyzing data safely and in accordance with robust regulatory standards and guidance. It is also crucial that these apps provide reliable information that clinicians can act on [98]. The context of the standards included in this study regarding data protection and security was to build mutual trust and maximize the benefits of eHealth information exchange. Trust is a key factor in the successful adoption and use of health apps, and transparency in data handling and clinical decision-making is essential to build and maintain that trust. This is also paramount for the widespread acceptance and impact of health apps on health care outcomes in sub-Saharan Africa.

We acknowledge the existence of numerous national laws related to data protection and security outside the health sector. Hence, guidelines that link these legislations together must be provided to ensure compliance with all relevant laws and guidance when using patient data. An example of how to achieve this is the United Kingdome’s guide to good practice for digital and data-driven health technologies that provides guidelines on how to abide by the laws and principles that govern data security and protection in the United Kingdom, including the GDPR, Data Protection Act 2018, and Caldicott Principles [105].

#### Standards and Interoperability

Standards and interoperability are essential for effectively developing, deploying, and implementing health apps to support self-management in sub-Saharan Africa. Interoperability is the ability of different systems, devices, or applications to communicate and exchange data with each other in a coordinated manner, thus providing timely and seamless portable information across organizational, regional, and national boundaries and optimizing both individual and population health [106]. In the same vein, standards enable interoperability between systems or devices through a common language and a common set of expectations [106].

Interoperability is crucial in improving the quality, safety, and efficiency of care delivery as well as empowering patients and providers with access to relevant and timely information [99]. One of the most widely used and accepted interoperability standards for health care data exchange is FHIR [106,107]. FHIR is a global industry standard developed by HL7 International. FHIR is designed to be quick to learn and implement and to support a variety of use cases, including self-management [108]. By using apps that are based on an FHIR standard, patients can benefit from data analytics that show how their health data relate to their chronic conditions or wellness goals [109]. They could also access all their health information from one place, even if they visit different health professionals who use different electronic medical records or EHR, thus promoting integrated care [28,31,33,109-115]. As a result, patient care can easily be coordinated.

The context of the included regulatory standards with regard to standards and interoperability was to address poor coordination, duplication of efforts, and inefficient use of resources and to promote the integration of ICT systems. However, in sub-Saharan Africa, there are many challenges and barriers to the adoption and implementation of interoperability standards, such as the lack of awareness or knowledge of the benefits and requirements of interoperability standards among stakeholders; lack of incentives or regulations to encourage or enforce the adoption of interoperability standards by app developers and vendors; lack of resources or capacity to implement interoperability standards, including technical expertise, infrastructure, funding, or governance; and lack of alignment or coordination among the different actors and initiatives involved in developing, deploying, and implementing the digital health interventions [30,116-119]. To address these challenges, some possible solutions may include raising awareness and education on the importance and value of interoperability standards for health apps among all relevant actors; developing and implementing policies and guidelines that promote or mandate the use of interoperability standards by app developers and vendors; providing technical assistance and support for app developers and vendors to adopt and implement interoperability standards, such as tools, frameworks, testing, certification, or accreditation; and establishing and strengthening collaboration and coordination among the different stakeholders and initiatives involved in health app development, deployment, and implementation in sub-Saharan Africa. In addition, the Digital Health Platform Handbook, a toolkit developed by the collaborative efforts of the WHO and ITU [120], can help countries in sub-Saharan Africa to develop and implement digital health platforms as the underlying infrastructure for interoperable and integrated national digital health systems. The digital health platform is a system-wide approach to developing digital health solutions with the aim to overcome the problems of siloed, vertical, and isolated applications and systems that hamper data management, innovation, efficiency, and impact in the health sector.

#### Inclusion and Equitable Access

Inclusion and equitable access are crucial to ensuring that health apps and related services are culturally appropriate and relevant as well as accessible to all who need them, regardless of gender, ethnicity, geographical location, ability, or financial status [24-29]. This is the key to promoting a “sense of belonging” and “ownership” and thus underscoring the importance of stakeholder mapping and involvement or engagement through the development and implementation process [22].

In this study, the included regulatory standards demonstrate the importance of inclusion by adopting both a participatory and consultative approach involving multiple stakeholders from different sectors. However, the standards do not provide clear guidance to ensure the adequate participation and sustained engagement of all relevant stakeholders. The lack of concise guidance to ensure the adequate participation and engagement of all relevant stakeholders, especially the susceptible and disadvantaged groups, can increase the risk of tokenistic tendencies, which can undermine the cultural appropriateness of health apps [25,121]. Some susceptible groups, such as women and people with low socioeconomic status, may face additional barriers to accessing and using health apps, such as lack of digital literacy, privacy concerns, cultural norms, or stigma [25]. Similarly, the cost of developing, maintaining, and updating health apps may not be covered by public or private health insurance schemes, which could limit their affordability and availability for low-income or uninsured populations [95]. However, there is no specific guidance or model for an effective funding mechanism for health apps in the included regulatory standards.

To address these challenges and ensure equitable access to health apps for self-management in sub-Saharan Africa, possible measures may include developing policies and regulations that support integrating health app interventions into existing health systems and financing mechanisms and engaging with stakeholders from different sectors and backgrounds (including health professionals, patients, communities, governments, civil society, academia, and industry) to co-develop and co-implement frameworks or models that promote the use of health apps for self-management in ways that are responsive to the local context and needs. Moreover, establishing regulations that provide appropriate financing or reimbursement options will reduce the risk of developers of good quality health apps turning to data mining for revenue, thus increasing privacy concerns [95]. For instance, in Germany, the reimbursement of health apps classified as medical devices (Digitale Gesundheitsanwendungen) was introduced in 2021 under the statutory health insurance [122,123]. When a medical device is prescribed by a physician or a physiotherapist, the manufacturer must submit an application to the German Federal Institute for Drugs and Medical Devices (Bundesinstitut für Arzneimittel und Medizinprodukte) for approval [123]. The Federal Association of the Statutory Health Insurance Funds (Spitzenverband Bund der Krankenkassen) determines and negotiates the reimbursement thresholds following approval. However, the manufacturer must demonstrate that the app is safe, functional, and of good quality; complies with data protection requirements; and benefits patient care [123].

#### Process

The process of regulating health apps essentially involves the development and implementation of regulatory standards and guidance. According to our study, the development process comprises a participatory and consultative process, a multisectoral approach, and a reference to international standards and best practices. In contrast, the implementation process is ongoing and requires appropriate legal authority, coordination, capacity building, and monitoring and evaluation.

We recognize that health apps can be accessed and used by patients from different parts of the world, and this means that countries need to carefully consider whether health apps that are accessed and used by their citizens meet the national or regional legal and ethical requirements, including their cultural and linguistic needs [23]. For countries in sub-Saharan Africa, a cross-border or regional collaboration between national legal authorities through the coordination of agencies such as the African Medicines Regulatory Harmonization (AMRH) may help to ensure that health apps built for the region are safe, effective, and user-friendly for everyone, considering the contextual differences of the countries [23]. For instance, all medical device companies that want to sell their products in the European market must obtain a Conformité Européenne (CE) mark for their devices, which indicates that they meet the legal requirements and can be freely circulated within the European Union [124]. Although the European Union member states regulate medical devices, the European Medicines Agency is involved in the regulatory process.

The regulation of health apps is extremely complex and involves a wide range of stakeholders. Therefore, a robust coordination mechanism is essential to reduce the risk of fragmentation and duplication of efforts and to promote the efficient use of resources. Most countries in sub-Saharan Africa have units in health ministries that coordinate and oversee the regulation of medical products. These units should be autonomous, full-fledged departments with legal authority (boards or commissions) to ensure independent, transparent, and accountable decision-making, but this is often not the case [125]. These units are recognized by the national authorities as regulators (eg, the National Medicines Regulatory Authority [NMRA]) [126]. Such organizational structures hinder the effectiveness of the national regulatory authorities in fulfilling their mandate and prevent the establishment of quality management systems to ensure transparent and accountable decision-making [125].

Furthermore, Essén et al [23] analyzed health app policy or regulation in 9 high-income countries (Sweden, Norway, Denmark, Netherlands, Belgium, Germany, England, the United States, and Singapore) and found that most of these countries adopted centralized approaches to app evaluation. Although centralized approaches might have advantages over self-evaluation, they may create bottlenecks and limit the availability of high-quality health apps for users. As suggested by Essén et al [23], a decentralized approach, such as the accreditation of evaluation agencies, maybe a worthwhile solution. However, this will require adequate coordination to ensure the consistency and reliability of the evaluation criteria and methods across different agencies as well as the transparency and accountability of the accreditation process. A possible way to achieve this is to adopt a common framework that can guide the evaluation and accreditation of health apps.

Similarly, the postmarket surveillance (PMS) system, which is a new regulation for medical devices in Europe, is a process of collecting and analyzing data on medical devices after they have been launched into the market to ensure their safety and performance and to identify any problems or need for improvements [127,128]. The PMS system is important because premarket data, which are obtained from testing a medical device before it is launched, have limitations in capturing the long-term performance and risks of the device [128]. Currently, the PMS system does not cover fitness and wellness apps, which are commonly used in self-management. Hence, Yu [93] proposed that the PMS system should also be applied to DHTs, such as fitness and wellness apps. They argue that the postmarket data would help regulators periodically review and adjust the regulatory standards for these groups of health apps based on their risks and benefits.

Drawing on the experience of the United Kingdom, it can be clearly demonstrated that the regulation of health apps is a complex, a multifaceted, and an evolving process that involves different regulators and criteria depending on the nature and function of the app. For instance, a centralized NHS Apps Library was launched as a beta site in April 2017 to provide patients with a collection of trusted and easy-to-use digital health tools [129]. The library provided access to a range of health apps that were reviewed and approved by the NHS, including apps that could help patients manage conditions such as diabetes, mental health, and chronic obstructive pulmonary disease [130]. However, the library was closed in December 2021 [131]. Although no reason for the closure was provided on the website, it is likely because of persistent concerns regarding the safety of patients and data privacy involving multiple apps including those listed in the library [12,14-16,131,132]. The NHS App was introduced in January 2019 before the closure of the NHS Apps Library to serve as the gateway for accessing NHS services including ordering repeat prescriptions and booking or managing appointments [133].

Furthermore, the United Kingdom Health Security Agency, formerly known as Public Health England, issued a guidance on criteria for health app assessment in October 2017 [99]. The purpose of this guidance was to ensure that all health apps built for the UK population work well and provide clear information about their functions, benefits, and intended outcomes for patients and health care professionals. On the basis of this guidance, those intending to build an app are required to conform to certain regulations before being considered for the app assessment process. The 2 main regulations are the medical device regulation and the Care Quality Commission (CQC) registration. Apps that are considered as medical devices must register with the MHRA and have a CE mark. Apps providing health or social care that fit into 1 of 14 regulated activities are required to register with the CQC before they can be assessed [134]. CQC is an independent regulator of health and social care services in England.

Similarly, the Organisation for the Review of Care and Health Apps (ORCHA) is a UK-based organization that independently evaluates and distributes health apps. It provides services such as app review, accreditation, curation, and recommendation within the United Kingdom and across the world [135]. ORCHA also enables organizations (including the NHS) to build a decentralized web-based digital health library of consumer-friendly over-the-counter apps [135-137]. These apps are continuously assessed by ORCHA against the standards and regulations in clinical and professional assurance, data quality and privacy, and usability and accessibility [137].

In addition, the Digital Technology Assessment Criteria (DTAC) were introduced in beta in October 2020, and its first official version was subsequently launched in February 2021 [138]. The DTAC plays a crucial role in ensuring that digital health tools meet the necessary standards in areas such as clinical safety, data protection, technical security, interoperability, usability, and accessibility. By serving as the national baseline criteria for DHTs in the NHS and social care, it provides a valuable framework for health care organizations during procurement. It also offers guidance for developers on the expectations for their digital technologies within the NHS and social care. This is an example of how a harmonized framework can help ensure the quality and safety of DHTs, including health apps.

In addition, the National Institute for Health and Care Excellence Evidence Standards Framework is a set of evidence standards for a wide range of DHTs designed to help evaluators and decision makers in the health care system to consistently identify DHTs that are likely to offer benefits to the users and the health care system [139]. The Evidence Standards Framework was first published in March 2019 and is ideally used before DHTs (including health apps) are considered for commissioning or procurement by the NHS [140]. It is a crucial tool for ensuring that DHTs are clinically effective and offer value to the health and care system in the United Kingdom. In August 2022, the framework was updated to include AI and data-driven technologies with adaptive algorithms [140].

Furthermore, DTx apps, which are a type of medical device, are not allowed into the UK market unless they comply with the UK GDPR and meet the requirements of DTAC. In addition, they must bear the CE or UK Conformity Assessed marks [141]. This means that DTx apps must demonstrate their safety and efficacy through clinical trials and comply with the relevant regulations for data protection and quality standards as regulated by the MHRA. DTx products are also recognized as DHTs under the National Institute for Health and Care Excellence Evidence Standards Framework [142]. DTx incorporates software to treat, prevent, or manage specific diseases or conditions [143,144]. The fact that DTx products typically focus on a narrow clinical indication and generate evidence of clinical efficacy underscores their potential to make a substantial contribution to self-management and health care delivery in general. The increasing recognition of the role of DTx in patient care by regulators is also noteworthy, and the creation of regulatory and reimbursement pathways for approved apps further enables DTx products to continue to play an important role in impacting health care delivery [1,143]. This is a testament to the potential of regulated health apps to revolutionize health care and improve patient outcomes.

Among the many lessons to learn from the experience of the United Kingdom is that the regulation of health apps must evolve to keep pace with advances in DHTs and adapt to the changing needs and demands of digital health. Moreover, efforts are being made to streamline the multifaceted approaches to simplify app regulation and access in the United Kingdom [23]. Therefore, a robust and dynamic coordination mechanism, along with political will, skilled personnel, reliable funding, and a robust framework for monitoring and evaluating progress and aligning key performance indicators, is essential for countries in sub-Saharan Africa to keep pace with the advancement in the regulation of health apps. There is also a need to strengthen collaboration and ensure regulatory harmonization among national regulatory authorities and continental bodies such as the regional economic communities, AMRH, and the WHO AFRO [126].

Capacity building and monitoring and evaluation are important factors for ensuring effective regulation of health apps given the complex nature of the process. The regulation of medical products (including health apps) in sub-Saharan Africa generally includes licensing and accreditation, evaluation, inspection, quality control, information dissemination and promotion, and monitoring of adverse events [125]. Therefore, high-level skills as well as effective monitoring and evaluation will be required to ensure the success of the process. For most countries in sub-Saharan Africa, the NMRA is responsible for coordinating and overseeing the regulatory system of medical products [125,126]. However, in most cases, NMRAs are unable to perform the core regulatory functions expected of them [145]. More than 90% of African countries have limited or no capacity to regulate medical products, with only 7% having moderately developed capabilities [145]. The lack of effective NMRAs in Africa exposes the citizens to potential harm by allowing unsafe, low-quality, and fake medical products to circulate and be used [145].

Although it is the responsibility of governments to establish functional regulatory systems and ensure effective monitoring and evaluation of the regulatory process, the involvement of international and continental organizations to support sub-Saharan African countries improve the regulatory capacity of their national regulatory agencies would be extremely beneficial. For instance, the African Medicines Agency (AMA) was established in November 2019 as a treaty adopted by the African Union Member States to help address the concerns arising from weak regulatory systems on the continent. At present, 37 countries have signed the AMA treaty, including 26 countries that have ratified it [146]. The main objective of the AMA is to enhance the capacity of States Parties and regional economic communities to regulate medical products to improve the quality, safety, and efficacy of medical products on the continent [147]. The AMA, in collaboration with other existing capacity building initiatives or organizations, such as the WHO Global Initiative on Digital Health, ITU, AMRH, WHO AFRO, and United Nations Children’s Fund, can assist sub-Saharan African countries in aligning their regulatory requirements with available resources and support them to acquire the necessary tools and skills to build effective and sustainable regulatory systems for health apps. This can be achieved by adopting a decentralized approach to engage a network of technical experts across the African Union similar to the model of the European Medicines Agency [148].

#### Actors or Stakeholders

The regulation of health apps often requires working with a wide range of actors or stakeholders. However, in this review, we identified only 2 main actor groups (those who provide digital health services and those who use the ICT infrastructure of the health ministry). These are the groups that are targeted by the included regulatory standards.

From a broader perspective, 12 categories of stakeholders according to their potential role in regulating health apps for the self-management were mapped in this study. The potential contribution of these stakeholders to the regulation of health apps for self-management in sub-Saharan Africa not only depends on their roles and responsibilities but also on their interests, needs, expectations, and influence [41,149-151]. Thus, a robust stakeholder analysis is paramount as it can help define the scope of the regulatory process, prioritize the requirements, manage the expectations, and ensure the engagement and participation of stakeholders throughout the regulatory process [41,152-156]. Our stakeholder mapping, as presented in Table 2 (refer to Multimedia Appendix 4 for more details), lays the foundation for national governments to conduct a robust stakeholder analysis and to adopt an all-inclusive stakeholder engagement strategy to manage and sustain the engagement and participation of all relevant stakeholders [157,158].

### Recommendations

Our review found that the regulation of health apps in sub-Saharan Africa is especially poor and almost nonexistent, as only Kenya has national standards that could address some of the regulatory issues related to health apps. Therefore, we recommend the following actions to help sub-Saharan African countries improve the regulation of health apps to support self-management:

- Establish a clear and consistent definition of what constitutes a health app (considering AI or machine learning) and what level of regulation is required for different types of apps.
- Develop and implement criteria and guidelines that ensure the quality, safety, and usability of health apps.
- Engage with independent app evaluators, such as ORCHA, to adopt a common framework that can guide the evaluation and accreditation of health apps and use the framework to create and maintain decentralized and transparent platforms that showcase and evaluate health apps for users and health care professionals.
- Develop and implement policies and regulations that enable sustainable funding for health apps such as integrating the use of health apps for self-management into existing health systems and financing pathways or mechanisms.
- Support and facilitate innovation and collaboration across the sub-Saharan Africa region, especially in areas including but not limited to data security and privacy, interoperability standards, usability, accessibility, funding, capacity building, and monitoring and evaluation of the regulatory process.
- Manage and sustain the engagement, involvement, and participation of all relevant stakeholders in the regulatory process by conducting a robust stakeholder analysis and adopting an all-inclusive stakeholder engagement strategy.

### Strengths and Limitations of the Study

This study has several strengths, which include an extensive search of gray literature and repositories, contact with key individuals, and the use of a systematic approach. Given that regulatory standards and guidance are unavailable in scientific databases, a wide range of gray literature and repositories were searched. In addition, contact was made with key staff members to obtain relevant documents, including those at the MOHs, the WHO country offices, and the WHO AFRO. Second, to enhance the strength of the study, a policy analysis framework was adapted and used to systematically organize the key study findings, whereas a deductive descriptive qualitative content analysis approach was used to identify and analyze texts that contained relevant concepts and other related information based on the 4 predefined themes. Third, the RISA tool was used to guide the mapping of key stakeholders. This has further increased the robustness of the study findings.

The limitations of this study include the fact that our literature search was conducted in English. Although the literature search was conducted in English, it yielded documents written in French and Portuguese from the ICTworks repository. Second, regulatory standards and guidance are not readily available on scientific databases; hence, it is possible that some relevant documents might have been missed. However, efforts were made to obtain these documents by contacting key stakeholders including key contact persons at the WHO AFRO, WHO country offices, and MOHs. In addition, contacting key individuals only for the purposes of requesting documents rather than conducting direct interviews was one of the limitations of this study. Interviewing key contact persons and stakeholders to obtain additional information could have strengthened the review; however, we did not interview any key individuals or stakeholders because it was beyond the scope of this review. Nonetheless, we recommend that future studies consider incorporating interviews to explore the perspectives of key stakeholders.

### Conclusions

Health apps are increasingly being used by patients to manage their health, and sub-Saharan African countries can leverage these apps to advance their progress toward achieving SDG 3 (good health and well-being) and UHC, especially given the rapid advancement of AI and big data. However, our study has established that the regulation of health apps in sub-Saharan Africa is inadequate to ensure that health apps are technically reliable and clinically safe; interoperable across systems; compliant with the principles of confidentiality of information and data privacy; culturally appropriate and relevant; and accessible to everyone regardless of gender, ethnicity, location, or income. Therefore, the region can learn from the experiences of some high-income countries such as the United Kingdom and Germany to develop and implement a robust and responsive regulatory system that supports the widespread adoption of safe, effective, and beneficial health apps for its population.

Following the publication of this review, a summary of the findings will be disseminated to the relevant organizations. In addition, the key findings will be summarized and presented at national, regional, and international conferences.

## Appendix

Multimedia Appendix 1 PRISMA-ScR (Preferred Reporting Items for Systematic Reviews and Meta-Analyses extension for Scoping Reviews) checklist.

Multimedia Appendix 2 Database search strategy.

Multimedia Appendix 3 Details of included documents.

Multimedia Appendix 4 Mapping of the stakeholders according to their potential role in regulating health apps for self-management.

## Abbreviations

AFRO: Regional Office for Africa
AI: artificial intelligence
AMA: African Medicines Agency
AMRH: African Medicines Regulatory Harmonization
CE: Conformité Européenne
CQC: Care Quality Commission
DHT: digital health technology
DTAC: Digital Technology Assessment Criteria
DTx: digital therapeutics
EHR: electronic health record
FHIR: Fast Healthcare Interoperability Resources
GDPR: General Data Protection Regulation
HL7: Health Level 7
ICT: information and communications technology
ITU: International Telecommunication Union
mHealth: mobile health
MHRA: Medicines and Healthcare products Regulatory Agency
MOH: Ministry of Health
NHS: National Health Service
NMRA: National Medicines Regulatory Authority
ORCHA: Organisation for the Review of Care and Health Apps
PMS: postmarket surveillance
PRISMA: Preferred Reporting Items for Systematic Reviews and Meta-Analyses
PRISMA-ScR: Preferred Reporting Items for Systematic Reviews and Meta-Analyses extension for Scoping Reviews
RISA: Reporting Items for Stakeholder Analysis
SDG: sustainable development goal
UHC: universal health coverage
WHO: World Health Organization

## Data Availability

The search strategy for PubMed, Scopus, and the World Health Organization AIM is presented in Multimedia Appendix 1. All data generated or analyzed during this study are included in this published article (and its supplementary information files). The documents analyzed are available directly from the relevant institutional websites, ICTworks repository [44] or upon request from the relevant government departments in each country. Additionally, documents in the list of references that are not accessible on the web can be solicited from the corresponding author on reasonable request.
