# Supplementary material for: Regulatory Standards and Guidance for the Use of Health Apps for Self-Management in Sub-Saharan Africa: Scoping Review
Source: J Med Internet Res. 2024 Apr 11;26:e49163. doi: 10.2196/49163 (PMC11046393; doi:10.2196/49163)
Supplement: Multimedia Appendix 2 [file jmir_v26i1e49163_app2.docx]

**Multimedia Appendix 2**

Database Search Strategy

Database: PubMed

Final search limited to January 2005 – January 2024

| ID | Search details | Results |
| --- | --- | --- |
| 1 | "polic*"[All Fields] OR "legislat*"[All Fields] OR "strateg*"[All Fields] OR "regulat*"[All Fields] OR "standard*"[All Fields] OR "criter*"[All Fields] OR "framework*"[All Fields] OR "guideline*" | 8, 344, 643 |
| 2 | "digital health"[MeSH Terms] OR ("digital"[All Fields] AND "health"[All Fields]) OR "digital health"[All Fields] OR ("telemedicine"[MeSH Terms] OR "telemedicine"[All Fields] OR "ehealth"[All Fields]) OR "e-Health"[All Fields] OR ("mhealth s"[All Fields] OR "telemedicine"[MeSH Terms] OR "telemedicine"[All Fields] OR "mhealth"[All Fields]) OR "m-Health"[All Fields] | 140, 787 |
| 3 | (("applicabilities"[All Fields] OR "applicability"[All Fields] OR "application"[All Fields] OR "applications"[All Fields] OR "applicative"[All Fields]) AND ("software"[MeSH Terms] OR "software"[All Fields] OR "software s"[All Fields] OR "softwares"[All Fields])) OR "app"[All Fields] OR (("software"[MeSH Terms] OR "software"[All Fields] OR "software s"[All Fields] OR "softwares"[All Fields]) AND "app"[All Fields]) OR (("mobile"[All Fields] OR "mobiles"[All Fields]) AND "app"[All Fields]) OR (("cell phone"[MeSH Terms] OR ("cell"[All Fields] AND "phone"[All Fields]) OR "cell phone"[All Fields] OR ("mobile"[All Fields] AND "phone"[All Fields]) OR "mobile phone"[All Fields]) AND "app"[All Fields]) OR (("medic"[All Fields] OR "medical"[All Fields] OR "medicalization"[MeSH Terms] OR "medicalization"[All Fields] OR "medicalizations"[All Fields] OR "medicalize"[All Fields] OR "medicalized"[All Fields] OR "medicalizes"[All Fields] OR "medicalizing"[All Fields] OR "medically"[All Fields] OR "medicals"[All Fields] OR "medicated"[All Fields] OR "medication s"[All Fields] OR "medics"[All Fields] OR "pharmaceutical preparations"[MeSH Terms] OR ("pharmaceutical"[All Fields] AND "preparations"[All Fields]) OR "pharmaceutical preparations"[All Fields] OR "medication"[All Fields] OR "medications"[All Fields]) AND "app"[All Fields]) | 108, 821 |
| 4 | "world health organisation"[All Fields] OR "world health organization"[MeSH Terms] OR ("world"[All Fields] AND "health"[All Fields] AND "organization"[All Fields]) OR "world health organization"[All Fields] OR ("WHO"[All Fields] AND ("african people"[MeSH Terms] OR ("african"[All Fields] AND "people"[All Fields]) OR "african people"[All Fields] OR "africans"[All Fields] OR "black people"[MeSH Terms] OR ("black"[All Fields] AND "people"[All Fields]) OR "black people"[All Fields] OR "african"[All Fields]) AND ("geographic locations"[MeSH Terms] OR ("geographic"[All Fields] AND "locations"[All Fields]) OR "geographic locations"[All Fields] OR "region"[All Fields] OR "region s"[All Fields] OR "regional"[All Fields] OR "regionalization"[All Fields] OR "regionalizations"[All Fields] OR "regionalize"[All Fields] OR "regionalized"[All Fields] OR "regionalizing"[All Fields] OR "regionally"[All Fields] OR "regionals"[All Fields] OR "regions"[All Fields])) OR ("africa"[MeSH Terms] OR "africa"[All Fields] OR "africa s"[All Fields] OR "africas"[All Fields]) OR ("Africa South of the Sahara"[MeSH Terms] OR ("africa"[All Fields] AND "south"[All Fields] AND "sahara"[All Fields]) OR "Africa South of the Sahara"[All Fields] OR ("sub"[All Fields] AND "saharan"[All Fields] AND "africa"[All Fields]) OR "sub saharan africa"[All Fields]) OR "Africa South of the Sahara"[All Fields] | 627, 083 |
| 5 | "algeria"[MeSH Terms] OR "algeria"[All Fields] OR ("angola"[MeSH Terms] OR "angola"[All Fields] OR "angola s"[All Fields]) OR ("benin"[MeSH Terms] OR "benin"[All Fields] OR "benin s"[All Fields]) OR ("botswana"[MeSH Terms] OR "botswana"[All Fields] OR "botswana s"[All Fields]) OR ("burkina faso"[MeSH Terms] OR ("burkina"[All Fields] AND "faso"[All Fields]) OR "burkina faso"[All Fields]) OR ("burundi"[MeSH Terms] OR "burundi"[All Fields]) OR ("cameroon"[MeSH Terms] OR "cameroon"[All Fields] OR "cameroons"[All Fields] OR "cameroon s"[All Fields]) OR "cape verde"[All Fields] OR "cabo verde"[All Fields] OR "central african republic"[All Fields] OR ("chad"[MeSH Terms] OR "chad"[All Fields]) OR ("comoros"[MeSH Terms] OR "comoros"[All Fields] OR "comoro"[All Fields]) OR "ivory coast"[All Fields] OR "cote d'ivoire"[All Fields] OR "democratic republic of congo"[All Fields] OR "equatorial guinea"[All Fields] OR ("eritrea"[MeSH Terms] OR "eritrea"[All Fields]) OR ("ethiopia"[MeSH Terms] OR "ethiopia"[All Fields] OR "ethiopia s"[All Fields]) OR ("gabon"[MeSH Terms] OR "gabon"[All Fields]) OR ("gambia"[MeSH Terms] OR "gambia"[All Fields] OR "gambia s"[All Fields]) OR ("ghana"[MeSH Terms] OR "ghana"[All Fields] OR "ghana s"[All Fields]) OR ("guinea"[MeSH Terms] OR "guinea"[All Fields] OR "guinea s"[All Fields] OR "guineas"[All Fields]) OR ("guinea bissau"[MeSH Terms] OR "guinea bissau"[All Fields] OR ("guinea"[All Fields] AND "bissau"[All Fields]) OR "guinea bissau"[All Fields]) OR ("kenya"[MeSH Terms] OR "kenya"[All Fields] OR "kenya s"[All Fields]) OR ("lesotho"[MeSH Terms] OR "lesotho"[All Fields]) OR ("liberia"[MeSH Terms] OR "liberia"[All Fields] OR "liberia s"[All Fields]) OR ("madagascar"[MeSH Terms] OR "madagascar"[All Fields] OR "madagascar s"[All Fields]) OR ("malawi"[MeSH Terms] OR "malawi"[All Fields] OR "malawi s"[All Fields]) OR ("mali"[MeSH Terms] OR "mali"[All Fields]) OR ("mauritania"[MeSH Terms] OR "mauritania"[All Fields]) OR ("mauritius"[MeSH Terms] OR "mauritius"[All Fields]) OR ("mozambique"[MeSH Terms] OR "mozambique"[All Fields] OR "mozambique s"[All Fields]) OR ("namibia"[MeSH Terms] OR "namibia"[All Fields] OR "namibia s"[All Fields]) OR ("niger"[MeSH Terms] OR "niger"[All Fields]) OR ("nigeria"[MeSH Terms] OR "nigeria"[All Fields] OR "nigeria s"[All Fields]) OR "republic of congo"[All Fields] OR ("congo"[MeSH Terms] OR "congo"[All Fields]) OR ("rwanda"[MeSH Terms] OR "rwanda"[All Fields] OR "rwanda s"[All Fields]) OR "sao tome and principe"[All Fields] OR (("saint s"[All Fields] OR "sainte"[All Fields] OR "saints"[MeSH Terms] OR "saints"[All Fields] OR "saint"[All Fields]) AND (thomas, and[Investigator] OR thomas and[Investigator]) AND ("prince"[All Fields] OR "princes"[All Fields])) OR ("senegal"[MeSH Terms] OR "senegal"[All Fields] OR "senegal s"[All Fields]) OR ("seychelles"[MeSH Terms] OR "seychelles"[All Fields]) OR "sierra leone"[All Fields] OR "south africa"[All Fields] OR "south sudan"[All Fields] OR ("eswatini"[MeSH Terms] OR "eswatini"[All Fields] OR "swaziland"[All Fields]) OR ("eswatini"[MeSH Terms] OR "eswatini"[All Fields]) OR ("togo"[MeSH Terms] OR "togo"[All Fields]) OR ("uganda"[MeSH Terms] OR "uganda"[All Fields] OR "uganda s"[All Fields]) OR ("tanzania"[MeSH Terms] OR "tanzania"[All Fields] OR "tanzania s"[All Fields]) OR ("zambia"[MeSH Terms] OR "zambia"[All Fields] OR "zambia s"[All Fields]) OR ("zimbabwe"[MeSH Terms] OR "zimbabwe"[All Fields] OR "zimbabwe s"[All Fields]) | 686, 806 |
| 6 | "world health organisation"[All Fields] OR "world health organization"[MeSH Terms] OR ("world"[All Fields] AND "health"[All Fields] AND "organization"[All Fields]) OR "world health organization"[All Fields] OR ("WHO"[All Fields] AND ("african people"[MeSH Terms] OR ("african"[All Fields] AND "people"[All Fields]) OR "african people"[All Fields] OR "africans"[All Fields] OR "black people"[MeSH Terms] OR ("black"[All Fields] AND "people"[All Fields]) OR "black people"[All Fields] OR "african"[All Fields]) AND ("geographic locations"[MeSH Terms] OR ("geographic"[All Fields] AND "locations"[All Fields]) OR "geographic locations"[All Fields] OR "region"[All Fields] OR "region s"[All Fields] OR "regional"[All Fields] OR "regionalization"[All Fields] OR "regionalizations"[All Fields] OR "regionalize"[All Fields] OR "regionalized"[All Fields] OR "regionalizing"[All Fields] OR "regionally"[All Fields] OR "regionals"[All Fields] OR "regions"[All Fields])) OR ("africa"[MeSH Terms] OR "africa"[All Fields] OR "africa s"[All Fields] OR "africas"[All Fields]) OR ("Africa South of the Sahara"[MeSH Terms] OR ("africa"[All Fields] AND "south"[All Fields] AND "sahara"[All Fields]) OR "Africa South of the Sahara"[All Fields] OR ("sub"[All Fields] AND "saharan"[All Fields] AND "africa"[All Fields]) OR "sub saharan africa"[All Fields]) OR "Africa South of the Sahara"[All Fields] OR ("algeria"[MeSH Terms] OR "algeria"[All Fields] OR ("angola"[MeSH Terms] OR "angola"[All Fields] OR "angola s"[All Fields]) OR ("benin"[MeSH Terms] OR "benin"[All Fields] OR "benin s"[All Fields]) OR ("botswana"[MeSH Terms] OR "botswana"[All Fields] OR "botswana s"[All Fields]) OR ("burkina faso"[MeSH Terms] OR ("burkina"[All Fields] AND "faso"[All Fields]) OR "burkina faso"[All Fields]) OR ("burundi"[MeSH Terms] OR "burundi"[All Fields]) OR ("cameroon"[MeSH Terms] OR "cameroon"[All Fields] OR "cameroons"[All Fields] OR "cameroon s"[All Fields]) OR "cape verde"[All Fields] OR "cabo verde"[All Fields] OR "central african republic"[All Fields] OR ("chad"[MeSH Terms] OR "chad"[All Fields]) OR ("comoros"[MeSH Terms] OR "comoros"[All Fields] OR "comoro"[All Fields]) OR "ivory coast"[All Fields] OR "cote d'ivoire"[All Fields] OR "democratic republic of congo"[All Fields] OR "equatorial guinea"[All Fields] OR ("eritrea"[MeSH Terms] OR "eritrea"[All Fields]) OR ("ethiopia"[MeSH Terms] OR "ethiopia"[All Fields] OR "ethiopia s"[All Fields]) OR ("gabon"[MeSH Terms] OR "gabon"[All Fields]) OR ("gambia"[MeSH Terms] OR "gambia"[All Fields] OR "gambia s"[All Fields]) OR ("ghana"[MeSH Terms] OR "ghana"[All Fields] OR "ghana s"[All Fields]) OR ("guinea"[MeSH Terms] OR "guinea"[All Fields] OR "guinea s"[All Fields] OR "guineas"[All Fields]) OR ("guinea bissau"[MeSH Terms] OR "guinea bissau"[All Fields] OR ("guinea"[All Fields] AND "bissau"[All Fields]) OR "guinea bissau"[All Fields]) OR ("kenya"[MeSH Terms] OR "kenya"[All Fields] OR "kenya s"[All Fields]) OR ("lesotho"[MeSH Terms] OR "lesotho"[All Fields]) OR ("liberia"[MeSH Terms] OR "liberia"[All Fields] OR "liberia s"[All Fields]) OR ("madagascar"[MeSH Terms] OR "madagascar"[All Fields] OR "madagascar s"[All Fields]) OR ("malawi"[MeSH Terms] OR "malawi"[All Fields] OR "malawi s"[All Fields]) OR ("mali"[MeSH Terms] OR "mali"[All Fields]) OR ("mauritania"[MeSH Terms] OR "mauritania"[All Fields]) OR ("mauritius"[MeSH Terms] OR "mauritius"[All Fields]) OR ("mozambique"[MeSH Terms] OR "mozambique"[All Fields] OR "mozambique s"[All Fields]) OR ("namibia"[MeSH Terms] OR "namibia"[All Fields] OR "namibia s"[All Fields]) OR ("niger"[MeSH Terms] OR "niger"[All Fields]) OR ("nigeria"[MeSH Terms] OR "nigeria"[All Fields] OR "nigeria s"[All Fields]) OR "republic of congo"[All Fields] OR ("congo"[MeSH Terms] OR "congo"[All Fields]) OR ("rwanda"[MeSH Terms] OR "rwanda"[All Fields] OR "rwanda s"[All Fields]) OR "sao tome and principe"[All Fields] OR (("saint s"[All Fields] OR "sainte"[All Fields] OR "saints"[MeSH Terms] OR "saints"[All Fields] OR "saint"[All Fields]) AND (thomas, and[Investigator] OR thomas and[Investigator]) AND ("prince"[All Fields] OR "princes"[All Fields])) OR ("senegal"[MeSH Terms] OR "senegal"[All Fields] OR "senegal s"[All Fields]) OR ("seychelles"[MeSH Terms] OR "seychelles"[All Fields]) OR "sierra leone"[All Fields] OR "south africa"[All Fields] OR "south sudan"[All Fields] OR ("eswatini"[MeSH Terms] OR "eswatini"[All Fields] OR "swaziland"[All Fields]) OR ("eswatini"[MeSH Terms] OR "eswatini"[All Fields]) OR ("togo"[MeSH Terms] OR "togo"[All Fields]) OR ("uganda"[MeSH Terms] OR "uganda"[All Fields] OR "uganda s"[All Fields]) OR ("tanzania"[MeSH Terms] OR "tanzania"[All Fields] OR "tanzania s"[All Fields]) OR ("zambia"[MeSH Terms] OR "zambia"[All Fields] OR "zambia s"[All Fields]) OR ("zimbabwe"[MeSH Terms] OR "zimbabwe"[All Fields] OR "zimbabwe s"[All Fields])) | 934,205 |
| 7 | ("polic*"[All Fields] OR "legislat*"[All Fields] OR "strateg*"[All Fields] OR "regulat*"[All Fields] OR "standard*"[All Fields] OR "criter*"[All Fields] OR "framework*"[All Fields] OR "guideline*"[All Fields]) AND ("digital health"[MeSH Terms] OR ("digital"[All Fields] AND "health"[All Fields]) OR "digital health"[All Fields] OR ("telemedicine"[MeSH Terms] OR "telemedicine"[All Fields] OR "ehealth"[All Fields]) OR "e-Health"[All Fields] OR ("mhealth s"[All Fields] OR "telemedicine"[MeSH Terms] OR "telemedicine"[All Fields] OR "mhealth"[All Fields]) OR "m-Health"[All Fields]) AND ((("applicabilities"[All Fields] OR "applicability"[All Fields] OR "application"[All Fields] OR "applications"[All Fields] OR "applicative"[All Fields]) AND ("software"[MeSH Terms] OR "software"[All Fields] OR "software s"[All Fields] OR "softwares"[All Fields])) OR "app"[All Fields] OR (("software"[MeSH Terms] OR "software"[All Fields] OR "software s"[All Fields] OR "softwares"[All Fields]) AND "app"[All Fields]) OR (("mobile"[All Fields] OR "mobiles"[All Fields]) AND "app"[All Fields]) OR (("cell phone"[MeSH Terms] OR ("cell"[All Fields] AND "phone"[All Fields]) OR "cell phone"[All Fields] OR ("mobile"[All Fields] AND "phone"[All Fields]) OR "mobile phone"[All Fields]) AND "app"[All Fields]) OR (("medic"[All Fields] OR "medical"[All Fields] OR "medicalization"[MeSH Terms] OR "medicalization"[All Fields] OR "medicalizations"[All Fields] OR "medicalize"[All Fields] OR "medicalized"[All Fields] OR "medicalizes"[All Fields] OR "medicalizing"[All Fields] OR "medically"[All Fields] OR "medicals"[All Fields] OR "medicated"[All Fields] OR "medication s"[All Fields] OR "medics"[All Fields] OR "pharmaceutical preparations"[MeSH Terms] OR ("pharmaceutical"[All Fields] AND "preparations"[All Fields]) OR "pharmaceutical preparations"[All Fields] OR "medication"[All Fields] OR "medications"[All Fields]) AND "app"[All Fields])) AND ("world health organisation"[All Fields] OR "world health organization"[MeSH Terms] OR ("world"[All Fields] AND "health"[All Fields] AND "organization"[All Fields]) OR "world health organization"[All Fields] OR ("WHO"[All Fields] AND ("african people"[MeSH Terms] OR ("african"[All Fields] AND "people"[All Fields]) OR "african people"[All Fields] OR "africans"[All Fields] OR "black people"[MeSH Terms] OR ("black"[All Fields] AND "people"[All Fields]) OR "black people"[All Fields] OR "african"[All Fields]) AND ("geographic locations"[MeSH Terms] OR ("geographic"[All Fields] AND "locations"[All Fields]) OR "geographic locations"[All Fields] OR "region"[All Fields] OR "region s"[All Fields] OR "regional"[All Fields] OR "regionalization"[All Fields] OR "regionalizations"[All Fields] OR "regionalize"[All Fields] OR "regionalized"[All Fields] OR "regionalizing"[All Fields] OR "regionally"[All Fields] OR "regionals"[All Fields] OR "regions"[All Fields])) OR ("africa"[MeSH Terms] OR "africa"[All Fields] OR "africa s"[All Fields] OR "africas"[All Fields]) OR ("Africa South of the Sahara"[MeSH Terms] OR ("africa"[All Fields] AND "south"[All Fields] AND "sahara"[All Fields]) OR "Africa South of the Sahara"[All Fields] OR ("sub"[All Fields] AND "saharan"[All Fields] AND "africa"[All Fields]) OR "sub saharan africa"[All Fields]) OR "Africa South of the Sahara"[All Fields] OR ("algeria"[MeSH Terms] OR "algeria"[All Fields] OR ("angola"[MeSH Terms] OR "angola"[All Fields] OR "angola s"[All Fields]) OR ("benin"[MeSH Terms] OR "benin"[All Fields] OR "benin s"[All Fields]) OR ("botswana"[MeSH Terms] OR "botswana"[All Fields] OR "botswana s"[All Fields]) OR ("burkina faso"[MeSH Terms] OR ("burkina"[All Fields] AND "faso"[All Fields]) OR "burkina faso"[All Fields]) OR ("burundi"[MeSH Terms] OR "burundi"[All Fields]) OR ("cameroon"[MeSH Terms] OR "cameroon"[All Fields] OR "cameroons"[All Fields] OR "cameroon s"[All Fields]) OR "cape verde"[All Fields] OR "cabo verde"[All Fields] OR "central african republic"[All Fields] OR ("chad"[MeSH Terms] OR "chad"[All Fields]) OR ("comoros"[MeSH Terms] OR "comoros"[All Fields] OR "comoro"[All Fields]) OR "ivory coast"[All Fields] OR "cote d'ivoire"[All Fields] OR "democratic republic of congo"[All Fields] OR "equatorial guinea"[All Fields] OR ("eritrea"[MeSH Terms] OR "eritrea"[All Fields]) OR ("ethiopia"[MeSH Terms] OR "ethiopia"[All Fields] OR "ethiopia s"[All Fields]) OR ("gabon"[MeSH Terms] OR "gabon"[All Fields]) OR ("gambia"[MeSH Terms] OR "gambia"[All Fields] OR "gambia s"[All Fields]) OR ("ghana"[MeSH Terms] OR "ghana"[All Fields] OR "ghana s"[All Fields]) OR ("guinea"[MeSH Terms] OR "guinea"[All Fields] OR "guinea s"[All Fields] OR "guineas"[All Fields]) OR ("guinea bissau"[MeSH Terms] OR "guinea bissau"[All Fields] OR ("guinea"[All Fields] AND "bissau"[All Fields]) OR "guinea bissau"[All Fields]) OR ("kenya"[MeSH Terms] OR "kenya"[All Fields] OR "kenya s"[All Fields]) OR ("lesotho"[MeSH Terms] OR "lesotho"[All Fields]) OR ("liberia"[MeSH Terms] OR "liberia"[All Fields] OR "liberia s"[All Fields]) OR ("madagascar"[MeSH Terms] OR "madagascar"[All Fields] OR "madagascar s"[All Fields]) OR ("malawi"[MeSH Terms] OR "malawi"[All Fields] OR "malawi s"[All Fields]) OR ("mali"[MeSH Terms] OR "mali"[All Fields]) OR ("mauritania"[MeSH Terms] OR "mauritania"[All Fields]) OR ("mauritius"[MeSH Terms] OR "mauritius"[All Fields]) OR ("mozambique"[MeSH Terms] OR "mozambique"[All Fields] OR "mozambique s"[All Fields]) OR ("namibia"[MeSH Terms] OR "namibia"[All Fields] OR "namibia s"[All Fields]) OR ("niger"[MeSH Terms] OR "niger"[All Fields]) OR ("nigeria"[MeSH Terms] OR "nigeria"[All Fields] OR "nigeria s"[All Fields]) OR "republic of congo"[All Fields] OR ("congo"[MeSH Terms] OR "congo"[All Fields]) OR ("rwanda"[MeSH Terms] OR "rwanda"[All Fields] OR "rwanda s"[All Fields]) OR "sao tome and principe"[All Fields] OR (("saint s"[All Fields] OR "sainte"[All Fields] OR "saints"[MeSH Terms] OR "saints"[All Fields] OR "saint"[All Fields]) AND (thomas, and[Investigator] OR thomas and[Investigator]) AND ("prince"[All Fields] OR "princes"[All Fields])) OR ("senegal"[MeSH Terms] OR "senegal"[All Fields] OR "senegal s"[All Fields]) OR ("seychelles"[MeSH Terms] OR "seychelles"[All Fields]) OR "sierra leone"[All Fields] OR "south africa"[All Fields] OR "south sudan"[All Fields] OR ("eswatini"[MeSH Terms] OR "eswatini"[All Fields] OR "swaziland"[All Fields]) OR ("eswatini"[MeSH Terms] OR "eswatini"[All Fields]) OR ("togo"[MeSH Terms] OR "togo"[All Fields]) OR ("uganda"[MeSH Terms] OR "uganda"[All Fields] OR "uganda s"[All Fields]) OR ("tanzania"[MeSH Terms] OR "tanzania"[All Fields] OR "tanzania s"[All Fields]) OR ("zambia"[MeSH Terms] OR "zambia"[All Fields] OR "zambia s"[All Fields]) OR ("zimbabwe"[MeSH Terms] OR "zimbabwe"[All Fields] OR "zimbabwe s"[All Fields]))) | 395 |
| 8 | (("polic*"[All Fields] OR "legislat*"[All Fields] OR "strateg*"[All Fields] OR "regulat*"[All Fields] OR "standard*"[All Fields] OR "criter*"[All Fields] OR "framework*"[All Fields] OR "guideline*"[All Fields]) AND ("digital health"[MeSH Terms] OR ("digital"[All Fields] AND "health"[All Fields]) OR "digital health"[All Fields] OR ("telemedicine"[MeSH Terms] OR "telemedicine"[All Fields] OR "ehealth"[All Fields]) OR "e-Health"[All Fields] OR ("mhealth s"[All Fields] OR "telemedicine"[MeSH Terms] OR "telemedicine"[All Fields] OR "mhealth"[All Fields]) OR "m-Health"[All Fields]) AND ((("applicabilities"[All Fields] OR "applicability"[All Fields] OR "application"[All Fields] OR "applications"[All Fields] OR "applicative"[All Fields]) AND ("software"[MeSH Terms] OR "software"[All Fields] OR "software s"[All Fields] OR "softwares"[All Fields])) OR "app"[All Fields] OR (("software"[MeSH Terms] OR "software"[All Fields] OR "software s"[All Fields] OR "softwares"[All Fields]) AND "app"[All Fields]) OR (("mobile"[All Fields] OR "mobiles"[All Fields]) AND "app"[All Fields]) OR (("cell phone"[MeSH Terms] OR ("cell"[All Fields] AND "phone"[All Fields]) OR "cell phone"[All Fields] OR ("mobile"[All Fields] AND "phone"[All Fields]) OR "mobile phone"[All Fields]) AND "app"[All Fields]) OR (("medic"[All Fields] OR "medical"[All Fields] OR "medicalization"[MeSH Terms] OR "medicalization"[All Fields] OR "medicalizations"[All Fields] OR "medicalize"[All Fields] OR "medicalized"[All Fields] OR "medicalizes"[All Fields] OR "medicalizing"[All Fields] OR "medically"[All Fields] OR "medicals"[All Fields] OR "medicated"[All Fields] OR "medication s"[All Fields] OR "medics"[All Fields] OR "pharmaceutical preparations"[MeSH Terms] OR ("pharmaceutical"[All Fields] AND "preparations"[All Fields]) OR "pharmaceutical preparations"[All Fields] OR "medication"[All Fields] OR "medications"[All Fields]) AND "app"[All Fields])) AND ("world health organisation"[All Fields] OR "world health organization"[MeSH Terms] OR ("world"[All Fields] AND "health"[All Fields] AND "organization"[All Fields]) OR "world health organization"[All Fields] OR ("WHO"[All Fields] AND ("african people"[MeSH Terms] OR ("african"[All Fields] AND "people"[All Fields]) OR "african people"[All Fields] OR "africans"[All Fields] OR "black people"[MeSH Terms] OR ("black"[All Fields] AND "people"[All Fields]) OR "black people"[All Fields] OR "african"[All Fields]) AND ("geographic locations"[MeSH Terms] OR ("geographic"[All Fields] AND "locations"[All Fields]) OR "geographic locations"[All Fields] OR "region"[All Fields] OR "region s"[All Fields] OR "regional"[All Fields] OR "regionalization"[All Fields] OR "regionalizations"[All Fields] OR "regionalize"[All Fields] OR "regionalized"[All Fields] OR "regionalizing"[All Fields] OR "regionally"[All Fields] OR "regionals"[All Fields] OR "regions"[All Fields])) OR ("africa"[MeSH Terms] OR "africa"[All Fields] OR "africa s"[All Fields] OR "africas"[All Fields]) OR ("Africa South of the Sahara"[MeSH Terms] OR ("africa"[All Fields] AND "south"[All Fields] AND "sahara"[All Fields]) OR "Africa South of the Sahara"[All Fields] OR ("sub"[All Fields] AND "saharan"[All Fields] AND "africa"[All Fields]) OR "sub saharan africa"[All Fields]) OR "Africa South of the Sahara"[All Fields] OR ("algeria"[MeSH Terms] OR "algeria"[All Fields] OR ("angola"[MeSH Terms] OR "angola"[All Fields] OR "angola s"[All Fields]) OR ("benin"[MeSH Terms] OR "benin"[All Fields] OR "benin s"[All Fields]) OR ("botswana"[MeSH Terms] OR "botswana"[All Fields] OR "botswana s"[All Fields]) OR ("burkina faso"[MeSH Terms] OR ("burkina"[All Fields] AND "faso"[All Fields]) OR "burkina faso"[All Fields]) OR ("burundi"[MeSH Terms] OR "burundi"[All Fields]) OR ("cameroon"[MeSH Terms] OR "cameroon"[All Fields] OR "cameroons"[All Fields] OR "cameroon s"[All Fields]) OR "cape verde"[All Fields] OR "cabo verde"[All Fields] OR "central african republic"[All Fields] OR ("chad"[MeSH Terms] OR "chad"[All Fields]) OR ("comoros"[MeSH Terms] OR "comoros"[All Fields] OR "comoro"[All Fields]) OR "ivory coast"[All Fields] OR "cote d'ivoire"[All Fields] OR "democratic republic of congo"[All Fields] OR "equatorial guinea"[All Fields] OR ("eritrea"[MeSH Terms] OR "eritrea"[All Fields]) OR ("ethiopia"[MeSH Terms] OR "ethiopia"[All Fields] OR "ethiopia s"[All Fields]) OR ("gabon"[MeSH Terms] OR "gabon"[All Fields]) OR ("gambia"[MeSH Terms] OR "gambia"[All Fields] OR "gambia s"[All Fields]) OR ("ghana"[MeSH Terms] OR "ghana"[All Fields] OR "ghana s"[All Fields]) OR ("guinea"[MeSH Terms] OR "guinea"[All Fields] OR "guinea s"[All Fields] OR "guineas"[All Fields]) OR ("guinea bissau"[MeSH Terms] OR "guinea bissau"[All Fields] OR ("guinea"[All Fields] AND "bissau"[All Fields]) OR "guinea bissau"[All Fields]) OR ("kenya"[MeSH Terms] OR "kenya"[All Fields] OR "kenya s"[All Fields]) OR ("lesotho"[MeSH Terms] OR "lesotho"[All Fields]) OR ("liberia"[MeSH Terms] OR "liberia"[All Fields] OR "liberia s"[All Fields]) OR ("madagascar"[MeSH Terms] OR "madagascar"[All Fields] OR "madagascar s"[All Fields]) OR ("malawi"[MeSH Terms] OR "malawi"[All Fields] OR "malawi s"[All Fields]) OR ("mali"[MeSH Terms] OR "mali"[All Fields]) OR ("mauritania"[MeSH Terms] OR "mauritania"[All Fields]) OR ("mauritius"[MeSH Terms] OR "mauritius"[All Fields]) OR ("mozambique"[MeSH Terms] OR "mozambique"[All Fields] OR "mozambique s"[All Fields]) OR ("namibia"[MeSH Terms] OR "namibia"[All Fields] OR "namibia s"[All Fields]) OR ("niger"[MeSH Terms] OR "niger"[All Fields]) OR ("nigeria"[MeSH Terms] OR "nigeria"[All Fields] OR "nigeria s"[All Fields]) OR "republic of congo"[All Fields] OR ("congo"[MeSH Terms] OR "congo"[All Fields]) OR ("rwanda"[MeSH Terms] OR "rwanda"[All Fields] OR "rwanda s"[All Fields]) OR "sao tome and principe"[All Fields] OR (("saint s"[All Fields] OR "sainte"[All Fields] OR "saints"[MeSH Terms] OR "saints"[All Fields] OR "saint"[All Fields]) AND (thomas, and[Investigator] OR thomas and[Investigator]) AND ("prince"[All Fields] OR "princes"[All Fields])) OR ("senegal"[MeSH Terms] OR "senegal"[All Fields] OR "senegal s"[All Fields]) OR ("seychelles"[MeSH Terms] OR "seychelles"[All Fields]) OR "sierra leone"[All Fields] OR "south africa"[All Fields] OR "south sudan"[All Fields] OR ("eswatini"[MeSH Terms] OR "eswatini"[All Fields] OR "swaziland"[All Fields]) OR ("eswatini"[MeSH Terms] OR "eswatini"[All Fields]) OR ("togo"[MeSH Terms] OR "togo"[All Fields]) OR ("uganda"[MeSH Terms] OR "uganda"[All Fields] OR "uganda s"[All Fields]) OR ("tanzania"[MeSH Terms] OR "tanzania"[All Fields] OR "tanzania s"[All Fields]) OR ("zambia"[MeSH Terms] OR "zambia"[All Fields] OR "zambia s"[All Fields]) OR ("zimbabwe"[MeSH Terms] OR "zimbabwe"[All Fields] OR "zimbabwe s"[All Fields])))) AND (2005:2024[pdat]) | 392 |

Database: **Scopus**

Final search limited to January 2005 to January 2024

| ID | Search details | Results |
| --- | --- | --- |
| 1 | ALL ( polic* OR legislat* OR strateg* OR regulat* OR standard* OR criter* OR framework* OR guideline* ) | 37,240,643 |
| 2 | ALL ( digital AND health OR ehealth OR e-health OR mhealth OR m-health ) | 935,069 |
| 3 | ALL ( application AND software OR app* OR software AND app* OR mobile AND app* OR mobile AND phone AND app* OR medical AND app* ) | 278,278 |
| 4 | ALL ( world AND health AND organization OR who AND african AND region OR africa OR sub-saharan AND africa OR "Africa South of the Sahara" ) OR ( algeria OR angola OR benin OR botswana OR burkina AND faso OR burundi OR cameroon OR "cape verde" OR "cabo verde" OR "central african republic" OR chad OR comoros OR "ivory coast" OR "cote d'ivoire" OR "democratic republic of congo" OR "equatorial guinea" OR eritrea OR ethiopia OR gabon OR gambia OR ghana OR guinea OR guinea-bissau OR kenya OR lesotho OR liberia OR madagascar OR malawi OR mali OR mauritania OR mauritius OR mozambique OR namibia OR niger OR nigeria OR "republic of congo" OR congo OR rwanda OR "são tomé and príncipe" OR "saint thomas and prince" OR senegal OR seychelles OR "sierra leone" OR "south africa" OR "south sudan" OR swaziland OR eswatini OR togo OR uganda OR tanzania OR zambia OR zimbabwe ) | 433,124 |
| 5 | ( ALL ( polic* OR legislat* OR strateg* OR regulat* OR standard* OR criter* OR framework* OR guideline* ) AND ALL ( digital AND health OR ehealth OR e-health OR mhealth OR m-health ) AND ALL ( application AND software OR app* OR software AND app* OR mobile AND app* OR mobile AND phone AND app* OR medical AND app* ) AND ALL ( world AND health AND organization OR who AND african AND region OR africa OR sub-saharan AND africa OR "Africa South of the Sahara" ) OR ( algeria OR angola OR benin OR botswana OR burkina AND faso OR burundi OR cameroon OR "cape verde" OR "cabo verde" OR "central african republic" OR chad OR comoros OR "ivory coast" OR "cote d'ivoire" OR "democratic republic of congo" OR "equatorial guinea" OR eritrea OR ethiopia OR gabon OR gambia OR ghana OR guinea OR guinea-bissau OR kenya OR lesotho OR liberia OR madagascar OR malawi OR mali OR mauritania OR mauritius OR mozambique OR namibia OR niger OR nigeria OR "republic of congo" OR congo OR rwanda OR "são tomé and príncipe" OR "saint thomas and prince" OR senegal OR seychelles OR "sierra leone" OR "south africa" OR "south sudan" OR swaziland OR eswatini OR togo OR uganda OR tanzania OR zambia OR zimbabwe ) ) AND PUBYEAR > 2004 | 2,399 |

Database: **WHO African Index Medicus**

Final search limited to January 2005 to January 2024

| ID | Search details | Results |
| --- | --- | --- |
| 1 | (tw:(Polic* OR legislat* OR strateg* OR regulat* OR standard* OR criter* OR framework* OR guideline*)) | 509,940 |
| 2 | (tw:(digital health OR ehealth OR e-health OR mhealth OR m-health)) | 26,413 |
| 3 | (tw:(application software OR app* OR software app* OR mobile app* OR mobile phone app* OR medical app*)) | 404 |
| 4 | (tw:(world health organization OR who african region OR africa OR sub-saharan africa OR "Africa South of the Sahara") OR (algeria OR angola OR benin OR botswana OR burkina faso OR burundi OR cameroon OR "cape verde" OR "cabo verde" OR "central african republic" OR chad OR comoros OR "ivory coast" OR "cote d'ivoire" OR "democratic republic of congo" OR "equatorial guinea" OR eritrea OR ethiopia OR gabon OR gambia OR ghana OR guinea OR guinea-bissau OR kenya OR lesotho OR liberia OR madagascar OR malawi OR mali OR mauritania OR mauritius OR mozambique OR namibia OR niger OR nigeria OR "republic of congo" OR congo OR rwanda OR "são tomé and príncipe" OR "saint thomas and prince" OR senegal OR seychelles OR "sierra leone" OR "south africa" OR "south sudan" OR swaziland OR eswatini OR togo OR uganda OR tanzania OR zambia OR Zimbabwe)) | 33,036 |
| 5 | tw:((tw:(polic* OR legislat* OR strateg* OR regulat* OR standard* OR criter* OR framework* OR guideline*)) AND (tw:(digital health OR ehealth OR e-health OR mhealth OR m-health)) AND (tw:(application software OR app* OR software app* OR mobile app* OR mobile phone app* OR medical app*)) AND (tw:(world health organization OR who african region OR africa OR sub-saharan africa OR "Africa South of the Sahara") OR (algeria OR angola OR benin OR botswana OR burkina faso OR burundi OR cameroon OR "cape verde" OR "cabo verde" OR "central african republic" OR chad OR comoros OR "ivory coast" OR "cote d'ivoire" OR "democratic republic of congo" OR "equatorial guinea" OR eritrea OR ethiopia OR gabon OR gambia OR ghana OR guinea OR guinea-bissau OR kenya OR lesotho OR liberia OR madagascar OR malawi OR mali OR mauritania OR mauritius OR mozambique OR namibia OR niger OR nigeria OR "republic of congo" OR congo OR rwanda OR "são tomé and príncipe" OR "saint thomas and prince" OR senegal OR seychelles OR "sierra leone" OR "south africa" OR "south sudan" OR swaziland OR eswatini OR togo OR uganda OR tanzania OR zambia OR zimbabwe))) AND (year_cluster:[2005 TO 2024]) | 1 |
