# Supplementary material for: Regulatory Standards and Guidance for the Use of Health Apps for Self-Management in Sub-Saharan Africa: Scoping Review
Source: J Med Internet Res. 2024 Apr 11;26:e49163. doi: 10.2196/49163 (PMC11046393; doi:10.2196/49163)
Supplement: Multimedia Appendix 3 [file jmir_v26i1e49163_app3.docx]

**Multimedia Appendix 3**

Details of included documents

| **S/no.** | **Standalone Regulatory Standards and Guidance Related to Health Apps**  **(Year of development and implementation)** | **Country (Language)** |
| --- | --- | --- |
| 1. | Standard for Electronic Health Record system (EHRs) in Ethiopia (2021) | Ethiopia (English) |
| 2 | Kenya Standards and Guidelines for mHealth Systems (2017) | Kenya (English) |
| 3. | Kenya Standards and Guidelines for E-Health Systems Interoperability (2014) |  |
| 4. | Health Sector ICT Standards and Guidelines (2013) |  |
| 5. | Standards and Guidelines for Electronic Medical Record Systems in Kenya (2020) |  |
| 6. | Health Information Exchange Standard Operating Procedure (SOP) and Guideline (2020) | Nigeria (English) |
| **S/no.** | **National Policies/Strategies on Digital Health or Health IT (Year of development and implementation)** | **Country (Language)** |
| 1. | National eHealth Strategy (2018-2022) | Benin (French) |
| 2. | The eHealth Strategy of Botswana (2020-2024) | Botswana (English) |
| 3. | Health Sector digital Strategy (2016-2020) | Burkina Faso (French) |
| 4. | National Health Informatics Development Plan of Burundi  (2015) | Burundi (French) |
| 5. | The 2020-2024 National Digital Health Strategic Plan (2020-2024) | Cameroon (English) |
| 6. | National eHealth Strategy (2017-2021) | Comoros (French) |
| 7. | eHealth Strategic Plan (2011) | Cote d’Ivoire or Ivory Coast (French) |
| 8. | National Development Plan for Health Informatics (2014) | Democratic Republic of the Congo (French) |
| 9. | Kingdom of Swaziland eHealth Strategy (2016-2020) | Eswatini (English) |
| 10. | Information Revolution Strategic Plan (2018-2025) | Ethiopia (English) |
| 11. | Strategic Master Plan of the Health Information System of Gabon (2017-2022) | Gabon (French) |
| 12. | National e-Health Strategy (2010) | Ghana (English) |
| 13. | Health Sector ICT Policy and Strategy (2005) |  |
| 14. | National eHealth Policy (2016-2030) | Kenya (English) |
| 15. | National e-Health Strategy (2011-2017) |  |
| 16. | Health Information System & ICT Strategic Plan (2016-2021) | Liberia (English) |
| 17. | Strategic Plan for Strengthening the Health Information System of Madagascar (2018–2022) | Madagascar (English) |
| 18. | National Digital Health Strategy 2020-2025 | Malawi (English) |
| 19. | National eHealth Policy in Mali (2013) | Mali (French) |
| 20. | He@lth 2015: Seamless Continuity of Care (2015) | Mauritius (English) |
| 21. | Strategic Plan of Information System for Health (2009-2014) | Mozambique (Portuguese) |
| 22. | National eHealth Strategy (2021-2025) | Namibia (English) |
| 23. | National eHealth Strategy (2019-2023) | Niger (French) |
| 24. | National Digital Health Strategy (2021 – 2025) | Nigeria (English) |
| 25. | National Digital Health Policy (2021) |  |
| 26. | National Digital Health Strategic Plan (2018-2023) | Rwanda (English) |
| 27. | Health Information System Strategic Plan (2018‐2023) | Senegal (French) |
| 28. | National Digital Health Strategy (2018 – 2023) | Sierra Leone (English) |
| 29. | National eHealth Strategy (2019-2024) | South Africa (English) |
| 30. | Tanzania National Digital Health Strategy (2019-2024) | Tanzania (English) |
| 31. | Strategic Plan for the Development of eHealth in Togo (2013-2015) | Togo (French) |
| 32. | Uganda National eHealth Strategy (2017-2021) | Uganda (English) |
| 33. | Uganda National eHealth Policy (2016) |  |
| 34. | National eHealth Strategy (2017-2021) | Zambia (English) |
| 35. | Zimbabwe’s E-Health Strategy (2012–2017) | Zimbabwe (English) |
| **S/no.** | **Other Related Documents**  **(Year of development and implementation)** | **Country (Language)** |
| 1. | Digital Health Blueprint (2021) | Ethiopia (English) |
| 2. | Kenya Health Information Systems Interoperability Framework (2020) | Kenya (English) |
| 3. | National Community Health Digitization Strategy (2020-2025) |  |
| 4. | Health Information System Interoperability in Liberia (2016) | Liberia (English) |
| 5. | Beyond National Digital Health Strategy: Final Report of End Term Evaluation for The National Health ICT Strategic Framework 2015-2020 (2021) | Nigeria (English) |
| 6 | Narrative for 2022 National Digital Health Annual Operational Plan (AOP) (2022) |  |
| 7. | 2021 National Health Normative Standards Framework for Digital Health Interoperability in South Africa (2021) | South Africa (English) |
| 8. | Tanzania Digital Health Investment Road Map ((2017-2023) | Tanzania (English) |
|  | |  |
