# Supplementary material for: Regulatory Standards and Guidance for the Use of Health Apps for Self-Management in Sub-Saharan Africa: Scoping Review
Source: J Med Internet Res. 2024 Apr 11;26:e49163. doi: 10.2196/49163 (PMC11046393; doi:10.2196/49163)
Supplement: Multimedia Appendix 4 [file jmir_v26i1e49163_app4.docx]

**Multimedia Appendix 4**

Mapping of the stakeholders according to their potential role in regulating health apps for self-management

| **Stakeholder category** | **List of stakeholders and examples** | **Role categorization** | **Role description** |
| --- | --- | --- | --- |
| A1 | - Government (health sector) - Ministry of Health - Relevant departments and agencies including the National Medicines Regulatory Authority | Coordination and provision of an enabling environment | - Oversee and coordinate the development and implementation of regulatory standards and guidance for the use of health apps for patient self-management at the national and state or regional levels and to ensure that they align with health goals and political support. - Establish a robust coordination mechanism that ensures accountability, transparency, and effective leadership of the regulatory process. - Promote awareness and engage relevant multisectoral stakeholders. - Understand stakeholders’ interests, level of influence, and potential contribution to the development and implementation of regulatory standards and guidance for the use of health apps for self-management. - Facilitate technical collaboration for the development and implementation of regulatory standards and guidance. - Adopt appropriate financial modelling and ensure financing is aligned with priorities while identifying government, donor and private sector funding to ensure that funding is sustainable. - Provide enabling environments (including physical infrastructure, services and systems) to promote the effective regulation of health apps to support self-management interventions. |
| A2 | - Government (non-health sector) - Ministry of Power or Energy - Ministry of Information and Communications Technology or Telecommunication - Ministry of Education - Ministry of Science and Technology - Ministry of Finance - Ministry of Justice | Coordination and provision of an enabling environment | - Provide enabling environments (including physical infrastructure, services and systems) to promote the effective regulation of health apps to support self-management interventions. |
| B | - Regulatory bodies - Relevant health regulatory agencies - Ministry of Justice - Law enforcement agencies | Compliance | - Create a legal environment to ensure compliance and enforcement of regulatory standards and guidance and thus establish trust and protection for patients and other users of health app for self-management. |
| C1 | - Funding bodies - Donors and aid agencies - Foundations and development banks - The private sector - Other health-care funders | Funding and insurance | - Provide funding required to develop and implement regulatory standards and guidance for the use of health apps for self-management. |
| C2 | - Insurance | Funding and insurance | - Provide funding required to develop and implement regulatory standards and guidance for the use of health apps for self-management. |
| D1 | - Intergovernmental, international, and continental organizations - African Union - WHOa/WHO Regional Office for Africa - International Telecommunication Union - World Bank - United Nations Children’s Fund | Strategic support | - Provide support (including technical and financial) to ensure effective regulation of health apps for self-management. |
| D2 | - Non-state actors - Nongovernmental organisations - Civil society organizations - Faith-based organizations |  | - Provide support (including technical and financial) to ensure effective regulation of health apps for self-management. |
| E1 | - Industries and businesses that influence the use of health apps - App developers - Network or internet providers - App evaluators | Resources and skills | - Support the regulation of health apps for self-management particularly in the area of development and evaluation of health apps to ensure that they are effective, safe, culturally functional and competent, and are accessible to those who need them regardless of gender, ethnicity, ability, or financial status. - Provide cost-effective services (including voice, SMS and data or internet services) to facilitate the regulation and adoption of health apps for self-management. |
| E2 | - Academia, and research bodies/institutions - Universities - Teaching hospitals - Research institutes | Resources and skills | - Provide skills and expertise to facilitate the effective regulation of health apps for self-management. - Generate evidence and increase access to current knowledge in order to improve and continuously update regulatory standards and guidance for the use of health apps for self-management. - Facilitate capacity building and knowledge transfer among regulatory bodies/agencies and other key stakeholders (e.g., healthcare community) to ensure effective regulation of health apps for self-management. |
| E3 | - Professionals in research and practice/subject matter experts | Resources and skills | - Provide skills and expertise to facilitate the effective regulation of health apps for self-management. - Generate evidence and increase access to current knowledge in order to improve and continuously update regulatory standards and guidance for the use of health apps for self-management. - Facilitate capacity building and knowledge transfer among regulatory bodies/agencies and other key stakeholders (e.g., healthcare community) to ensure effective regulation of health apps for self-management. |
| F1 | - The health care community (providers) - Health care providers (e.g., hospitals, clinics, and primary health centres) - Health care professionals | Service delivery and use | - Contribute to the development and implementation of regulatory standards and guidance for the use of health apps for self-management interventions particularly to promote inclusion and equitable access. - Contribute to ensuring compliance to regulatory standards and guidance for the use of health apps for self-management. - Provide feedback that can be used to improve and update regulatory standards and guidance for the use of health apps for self-management. |
| F2 | - The health care community (users) - Patients - Caregivers - Families - Community groups | Service delivery and use | - Contribute to the development and implementation of regulatory standards and guidance for the use of health apps for self-management interventions particularly to promote inclusion and equitable access. - Contribute to ensuring compliance to regulatory standards and guidance for the use of health apps for self-management. - Provide feedback that can be used to improve and update regulatory standards and guidance for the use of health apps for self-management. |

^a^WHO: World Health Organization
